# IDO1-AhR axis increases T regulatory cells in *Plasmodium vivax* malaria infection

**DOI:** 10.3389/fimmu.2025.1474447

**Published:** 2025-07-14

**Authors:** Rafaella Oliveira dos Santos, Nani Oliveira Carvalho, Thiago Barros do Nascimento de Morais, Wlademir Braga Salgado Sobrinho, Maria Geuziane Soares da Cruz, Elaine Speziali de Faria, Fernanda Fortes de Araújo, Vanessa Peruhype Magalhães, Stefanie Costa Pinto Lopes, Adriana Malheiros, Adolfo José da Mota, Helton da Costa Santiago, Paulo Afonso Nogueira, Andréa Teixeira de Carvalho, Marcus Vinícius Guimarães de Lacerda, Pritesh Lalwani

**Affiliations:** ^1^ Instituto Leônidas e Maria Deane (ILMD), Fiocruz Amazônia, Manaus, Amazonas, Brazil; ^2^ Laboratory of Infectious Diseases and Immunology, Instituto Leônidas e Maria Deane, Fiocruz Amazônia and Programa de Pós-Graduação Stricto Sensu em Imunologia Básica e Aplicada, Manaus, Brazil; ^3^ Instituto René Rachou (IRR), Fiocruz Minas Gerais, Belo Horizonte, Minas Gerais, Brazil; ^4^ Instituto de Ciências Biológicas, Universidade Federal do Amazonas (UFAM), Manaus, Amazonas, Brazil; ^5^ Instituto de Ciências Biológicas, Universidade Federal de Minas Gerais (UFMG), Belo Horizonte, Minas Gerais, Brazil; ^6^ Fundação de Medicina Tropical, Dr. Heitor Vieira Dourado (FMT-HVD), Manaus, Amazonas, Brazil

**Keywords:** *Plasmodium vivax*, malaria, indoleamine 2,3-dioxygenase, tryptophan catabolism, kynurenine, regulatory T cells, immune tolerance, aryl hydrocarbon receptor

## Abstract

**Introduction:**

Malaria remains a significant public health challenge in Brazil, where *Plasmodium vivax* (*P. vivax*) is the predominant species. Dysregulated immune responses contribute substantially to malaria pathogenesis. Indoleamine 2,3-dioxygenase (IDO) mediates the catabolism of tryptophan (TRP) into kynurenine (KYN), an immunosuppressive metabolite implicated in immune tolerance. This study aimed to investigate the role of TRP catabolism and regulatory T cells (Tregs) during *P. vivax* infection.

**Methods:**

Peripheral blood mononuclear cells (PBMCs) were stimulated in vitro with *P. vivax*-infected erythrocyte (Pv-iE) lysate to assess IDO-1 expression, KYN/TRP ratio, cytokine production, and Treg frequency. The effects of pharmacological inhibition of IDO, MyD88, and aryl hydrocarbon receptor (AhR) pathways were evaluated. Additionally, plasma KYN/TRP ratio, Treg frequencies, and cytokine levels were measured in patients with acute *P. vivax* infection and compared between individuals experiencing their first malaria episode and those with previous infections.

**Results:**

Stimulation with Pv-iE lysate increased IDO-1 expression in CD14^+^ cells, elevated KYN/TRP ratio, and induced pro-inflammatory cytokine production. IDO inhibition reduced KYN/TRP ratio and Treg frequencies upon Pv-iE stimulation. MyD88 inhibition decreased both IDO-1 expression and KYN/TRP ratio. IDO and AhR inhibition reduced Treg frequencies and CD4^+^ T cell proliferation. Patients with acute *P. vivax* malaria exhibited elevated KYN/TRP ratios and increased Treg frequencies, with a positive correlation between these parameters. Individuals with prior malaria episodes showed lower Treg frequencies, plasma IFN-γ, and KYN/TRP ratios compared to those with primary infections.

**Discussion:**

These findings highlight the role of IDO-mediated TRP catabolism and innate immune signaling in promoting a tolerogenic phenotype during *P. vivax* infection. The study provides novel insights into mechanisms that may contribute to immune regulation, chronic inflammation, and tolerance during malaria, with potential implications for therapeutic interventions.

## Introduction

*Plasmodium vivax* is a less studied form of the malaria parasite that infects over 20 million people each year. *P. vivax* is the predominant species in Brazil, accounting for more than 80% of malaria infections. Most of these episodes are confined to the Amazon region, with isolated cases occurring in other states of the country (1, 2). Additionally, dormant liver stage hypnozoites that can be activated several weeks or months or even years after primary infection impose a serious challenge for malaria vivax treatment and elimination (3). Although *P. vivax* malaria infections present lower parasitic loads and rare complicated cases, several recent studies have reinforced the association between severe disease and death in *P. vivax* infections (4, 5).

For the efficient control of *Plasmodium* infections in the liver and blood stages of the parasite life cycle, the host immune system employs a strong pro-inflammatory immune response that must be balanced with regulatory mechanisms to avoid immunopathology. Indeed the immune system utilizes a variety of mechanisms to regulate the immune system during acute infections. For example, the enzyme indoleamine 2,3-dioxygenase (IDO) catabolizes tryptophan (TRP), an essential amino acid, into an immunosuppressive metabolite kynurenine (KYN), which binds to the aromatic hydrocarbon receptor (Ahr), promoting the differentiation of naive CD4+ T cells into regulatory CD4+FOXP3+ T cells (Tregs) and effector T cell suppression, anergy, and death (6, 7). In malaria, Tregs frequencies have been shown to increase in human and mouse infections, which have been shown to delay parasite clearance and decrease effector T cell function (8, 9). Tregs play an important role in maintaining a delicate balance between immunopathology and parasite clearance, but little is known about the mechanisms of Treg induction during acute *P. vivax* malaria. Hence, in this study, we investigated the relationship between innate immune activation, tryptophan catabolism, and regulatory T cell generation after *P. vivax* malaria infection.

## Materials and methods

### Ethics statement

This study was approved by the Universidade Federal do Amazonas (UFAM, CAAE: 36936614.3.0000.5020). All of the study participants provided an informed consent prior to enrollment.

### Study population

A cross-sectional cohort of patients infected with *P. vivax* was performed in the city of Manaus, Amazonas state. Samples were collected at the Fundação de Medicina Tropical Dr. Heitor Vieira Dourado (FMT-HVD) between June 2015 and January 2018. *Plasmodium* infection was confirmed and characterized by thick blood smear microscopy, with differential parasitemia count (sexual and asexual stages) in 200 leukocytes. The study participants were divided into three groups: (1) positive for *P. vivax* (Pv) with first malaria episode (*n* = 14), (2) positive individuals with previous malaria infection (*n* = 9), and (3) healthy endemic controls without symptoms and negative for malaria (*n* = 11) as previously described (10). The signs and symptoms among malaria cases ranged from very mild to more complicated symptoms; however, none of the patients presented disease severity or were hospitalized.

### Blood cell count

About 10 mL of venous blood was collected in EDTA tubes (BD Vacutainer). Initially, a complete blood count of the blood samples was performed using an automated hematological analyzer (Sysmex). The remaining plasma and PBMCs were separated and frozen at -80°C.

### Measurement of *P. vivax* lactate dehydrogenase

Plasma samples of vivax-positive and vivax-negative patients were analyzed by using sandwich ELISA to detect PvLDH (Sousa et al., 2014). In summary, the ELISA plates were coated with polyclonal rabbit anti-PvLDH antibodies. Subsequently, the patients’ plasma samples were added to detect free PvLDH. The ELISA was developed using a secondary anti-mouse IgG antibody conjugated with enzyme horseradish peroxidase (HRP). The cutoff values were calculated by the mean of the negative samples plus two standard deviations.

### PBMC separation and storage

Peripheral mononuclear blood cells (PMBCs) were separated using density gradient centrifugation with Ficoll Plaque-Plus reagent (GE Life Science) from malaria-naïve patients for *in vitro* stimulation. First, the blood was diluted in PBS-1X (1:1) and transferred to conical tubes containing Ficoll; the diluted blood to Ficoll ratio was 2:1. These tubes were then centrifuged for 35 min at 450*g* at 25°C, without break. After centrifugation, the PBMCs were collected and the cells were washed twice with PBS-1X. Then, the cell pellet was resuspended, and samples from all individuals were frozen (90% FCS + 10% DMSO) in liquid nitrogen until further analysis.

### *P. vivax*-infected red cell lysate separation and preparation

Blood was collected from *P. vivax*-infected patients with mature stage of the parasite (trophozoite and schizont) visualized in the thick blood smear for isolation of infected erythrocytes. First, the blood was washed three times with RPMI medium. Then, it was applied to a column with cellulose to separate the RBCs from the leukocytes and platelets. After filtration, the RBC pellet was washed three times with RPMI, then resuspended in RPMI medium, and added to a tube containing 45% Percoll (45% Percoll, 45% filtered H_2_O, and 10% RPMI 10X). These tubes were then centrifuged for 15 min at 1,500 RPM at 25°C to separate iPV-RBC. After centrifugation, the parasite-containing layer was removed and transferred to another tube. The pellet was then washed with RPMI three times. A thick blood smear was performed to count iPV-RBC. Each preparation of the infected erythrocyte extract was made up of a pool with the iPV-RBC from five to 10 patients. The microtubes containing the iPV-RBC were frozen–thawed three times for total RBC lysis, before use. As negative control, we used the same number of uninfected erythrocyte lysate (uRBC) and performed freeze–thaw before use.

### PBMC culture with iPV-RBC lysate

Malaria-naïve PBMCs at 1 × 10^6^ were cultured in RPMI 1640 (Gibco) with 10% fetal bovine serum (Gibco) and 1% penicillin–streptomycin (Life Technologies) in 24- and 96- well plates for a maximum of 6 days in an incubator at 37°C with 5% CO_2_. The IDO enzyme was inhibited with 1-methyl-D-tryptophan (1-MT) (Ref: 452483; Lot: MKBQ3449V; SIGMA-ALDRICH) at a concentration of 1 mM, and methanol was used as a negative control. MyD88 adapter protein was inhibited using a peptide (DRQIKIWFQNRRMKWKKRDVLPGT) (GenOne, Rio de Janeiro, Brazil) that mimics the protein portion, preventing homodimerization and interfering with its interaction at the MyD88-TIR domain, and a control peptide (DRQIKIWFQNRRMKWKK) as a negative control (GenOne). To block AhR, we used an antagonist, CH-223191, at a concentration of 10 mM and DMSO as a negative control.

### Cytokine quantification

The cytokine dosage of the patient’s plasma samples and cell culture supernatant was performed using the BD™ Human T_H_1, T_H_2 e T_H_17 CBA Kit, following the manufacturer’s guidelines. The samples were analyzed on FACSCanto or FACSVerse flow cytometers (Becton, Dickinson and Company, San Jose, CA, USA). The fluorescence intensity of the samples was correlated to the standard curve for each cytokine and expressed as pg/mL. CBA data was analyzed by using FCAP Array™ software (V3.0.1).

### Identification and quantification of tryptophan (TRP) and kynurenine

The patients’ plasma and cell culture supernatants (200 μL) were subjected to treatment with 8% perchloric acid for protein precipitation and analyzed by using high-performance liquid chromatography (HPLC) for TRP and KYN identification as previously described (10, 11).

### Cellular immunophenotyping

Immunophenotypic characterization was performed after *in vitro* stimulation of malaria-naïve PBMCs and *ex vivo* PBMC isolation from acutely endemic healthy controls and *P. vivax*-infected patients. Frozen PBMCs were thawed, washed, counted, and rested overnight in RPMI media supplemented as previously published at 37°C and 5% CO_2_. The thawed cells were only processed if ≥85% viable using trypan blue exclusion. Between 1.5 × 10^5^ and 2 × 10^5^ PBMC cells from healthy controls and infected patients were used for staining, with a minimum acquisition of 50,000 events per sample. For the *in vitro* experiments, 1 × 10^6^ cells were stimulated, with at least 100,000 events acquired. For staining, cells were incubated with surface antibodies for 30 min. The samples were washed with FACS buffer and resuspended in FACS lysing solution containing paraformaldehyde for pre-fixation. The cells were then washed again with FACS buffer, resuspended in FACS permeabilizing solution (BD Biosciences), and stained for intracellular markers (**Supplementary Table S1**) for 45 min. *In vitro* experiments were analyzed using the FACSCanto flow cytometer, and *ex vivo* patient PBMCs were analyzed on LSRFortessa X-20 flow cytometer (Becton, Dickinson and Company, San Jose, CA, USA). The flow cytometers used were calibrated daily by the core facility staff using the manufacturer’s cytometer settings and tracking calibration software, and identical voltages are used for all acquisitions for all fluorescent channels on all samples. All flowcytometry data was analyzed using the FlowJo™ 10.0 software (**Supplementary Tables S1**, **S3**, **Supplementary Figures 11**, **12**).

### RNA extraction and qPCR

RNA was extracted and purified using the RNeasy Mini Kit (Qiagen), following the supplier’s protocol. Purified RNA was suspended in 30 µL, and RNA yield was determined for each sample by Nanodrop. To prevent genomic DNA contamination, an additional step of residual DNA digestion was performed with DNase I (Ambion, 2 U/µL) for 30 min at 37°C, with further inactivation of the enzyme by incubating the samples at 75°C for 10 min. Next, we added EDTA (5 mM) to preserve RNA integrity. cDNA synthesis was performed using the High-Capacity cDNA Reverse Transcriptase Kit (Applied Biosystems) supplemented with additional MgCl_2_. The qPCR cycles were performed following the manufacturer’s instruction. For quantitative PCR (qPCR), to measure the gene expression of IDO1 and IDO2, qPCR was carried out using a QuantStudio 6 Flex Real-Time PCR System. GAPDH, ACTB, and B2M were chosen as normalizing genes for this experiment. In short, on a 96-well plate, 1 µL of cDNA sample was added to each well containing qPCR mix for a total of three replicates per sample. The qPCR mix was prepared with 5 µL of Power SYBR Green Master Mix (ThermoFisher), 0.2 µL of each primer (forward and reverse), and 3.6 µL of DEPC-treated water per reaction. Additionally, the plates also contained three negative control replicates for each gene that was analyzed. The primers’ sequences are described in **Supplementary Table S2**.

### Statistical analysis

The data was analyzed using GraphPad Prism software v9.5.0 (GraphPad Software, La Jolla, CA, USA). Normality was assessed using tests such as Shapiro–Wilk or D’Agostino–Pearson. Since the data did not follow a normal distribution, non-parametric tests were applied: Mann–Whitney *U*-test for two-group comparisons and Kruskal–Wallis for multiple group comparisons. Additionally, a two-way ANOVA was performed for analyses involving two independent variables. Correlations between variables were determined using Pearson’s correlation coefficient. A *p*-value of <0.05 was considered statistically significant for all tests.

## Results

### *In vitro Plasmodium vivax* stimulation of PBMCs increases tryptophan catabolism to kynurenine via the MyD88 pathway

**Figure 1:**
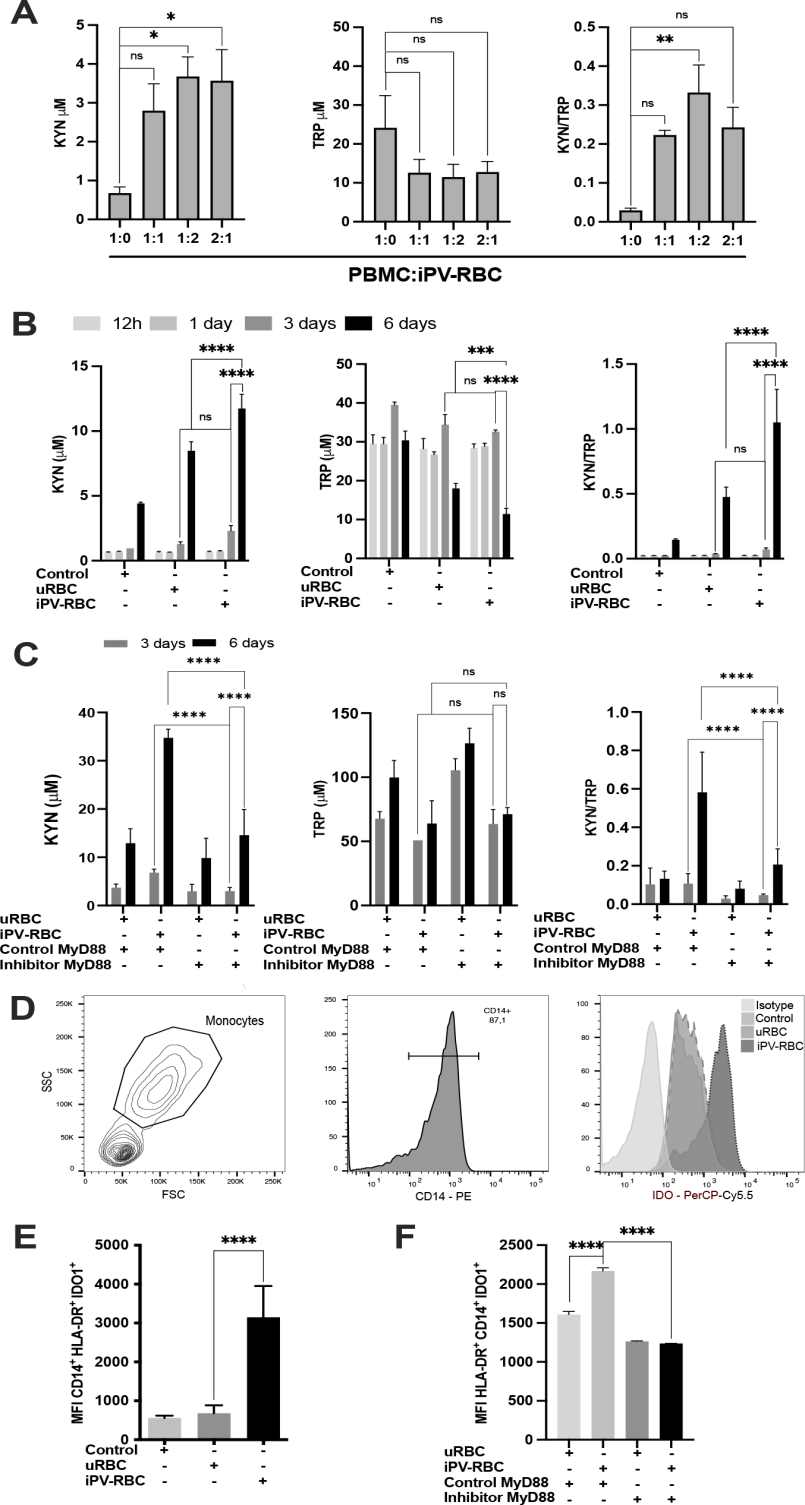
Stimulation with *Plasmodium vivax*-infected erythrocytes (iPV-RBC) through the MyD88 pathway leads to elevated kynurenine metabolites and increased expression of the indoleamine 2,3-dioxygenase (IDO) enzyme. *In vitro* healthy malaria-naïve donor PBMCs were stimulated with freeze–thaw lysate of *Plasmodium vivax*-infected erythrocytes. Cell culture supernatants were used to quantify tryptophan (TRP) and kynurenine (KYN) levels. **(A)** PBMCs were left unstimulated or stimulated with different ratios of iPV-RBCs for 3 days. **(B)** PBMCs were left unstimulated as controls in RPMI1640 and 10% FCS, stimulated with uninfected RBCs (uRBC) at 1:2 ratio, or stimulated with iPV-RBCs at 1:2 ratio. TRP and KYN was measured in cell culture supernatant at different time points. **(C)** TRP and KYN was measured in cell supernatant measured in PBMCs post-stimulation in the presence of control peptide or inhibitory peptide (26 amino acid) that blocks MyD88 signaling by inhibiting its homodimerization. Stimulated PBMCs were stained 3 days post-stimulation for CD14 and intracellular IDO1 protein expression. The cells were analyzed by flow cytometry, and IDO1 expression is represented as **(D)** histogram in CD14+ cells. **(E)** IDO1 expression is expressed as MFI in CD14+HLA-DR+ cells after stimulation in the **(F)** presence or absence of MyD88 inhibitor. The data are shown as mean + SD of one representative donor PBMC stimulated in quadruplicate. The experiment was performed with three independent malaria-naïve donors. Kruskal–Wallis with Dunn's multiple-comparisons test was used for **(A)**, **(C, D, F)**. Two-way ANOVA with Tukey's multiple-comparisons test was used for **(B, E)**. NS, not significant. *p < 0.05, **p < 0.01, ***p < 0.001, and ****p < 0.0001.

We verified if *P. vivax* malaria modulates tryptophan catabolism by stimulating peripheral blood mononuclear cells (PBMCs) from six healthy malaria-naive donors with uninfected red blood cells (uRBCs) or *P. vivax*-infected RBC (iPV-RBC) preparations. Initially, we stimulated PBMCs with a different ratio of iPV-RBC (**Figure 1A**) to identify the ideal PBMC-to-parasite ratio for these experiments. We could determine that 1:2 PBMC to iPV-RBC ratio was able to induce high catabolism of KYN that depleted TRP. We also tested different incubation times (**Figure 1B**), and we observed a gradual decrease in TRP and an increase in KYN metabolite in the culture supernatant compared to the negative control or uRBC groups at day 3 post-stimulation, which peaked at day 6 (**Figure 1B**). Next, we evaluated the role of Toll-like Receptors (TLRs) in *P. vivax*-stimulated IDO1 expression, we stimulated PBMC with iPV-RBC in presence of MyD88 inhibitory or control peptide. MyD88 inhibition significantly decreased *P. vivax*-induced TRP catabolism decreasing KYN/TRP ratio (**Figure 1C**). The increase in KYN/TRP ration at day 3 was associated with a significant increase in IDO1 protein expression in CD14+HLA-DR+ cell populations upon iPV-RBC stimulation (**Figures 1D, E**), whereas, MyD88 inhibition decreased IDO1 protein expression in CD14+HLA-DR+ cells (**Figure 1F**). iPV-RBC stimulation also significantly increased IDO1 gene expression on day 3 post stimulation, but not on day 6 (**Supplementary Figures 1A, B**). IDO2 gene expression was not significantly altered after iPV-RBC stimulation (**Supplementary Figures 1C, D**).

### IDO1 and AhR are essential for regulatory T cell (Treg) expansion

**Figure 2:**
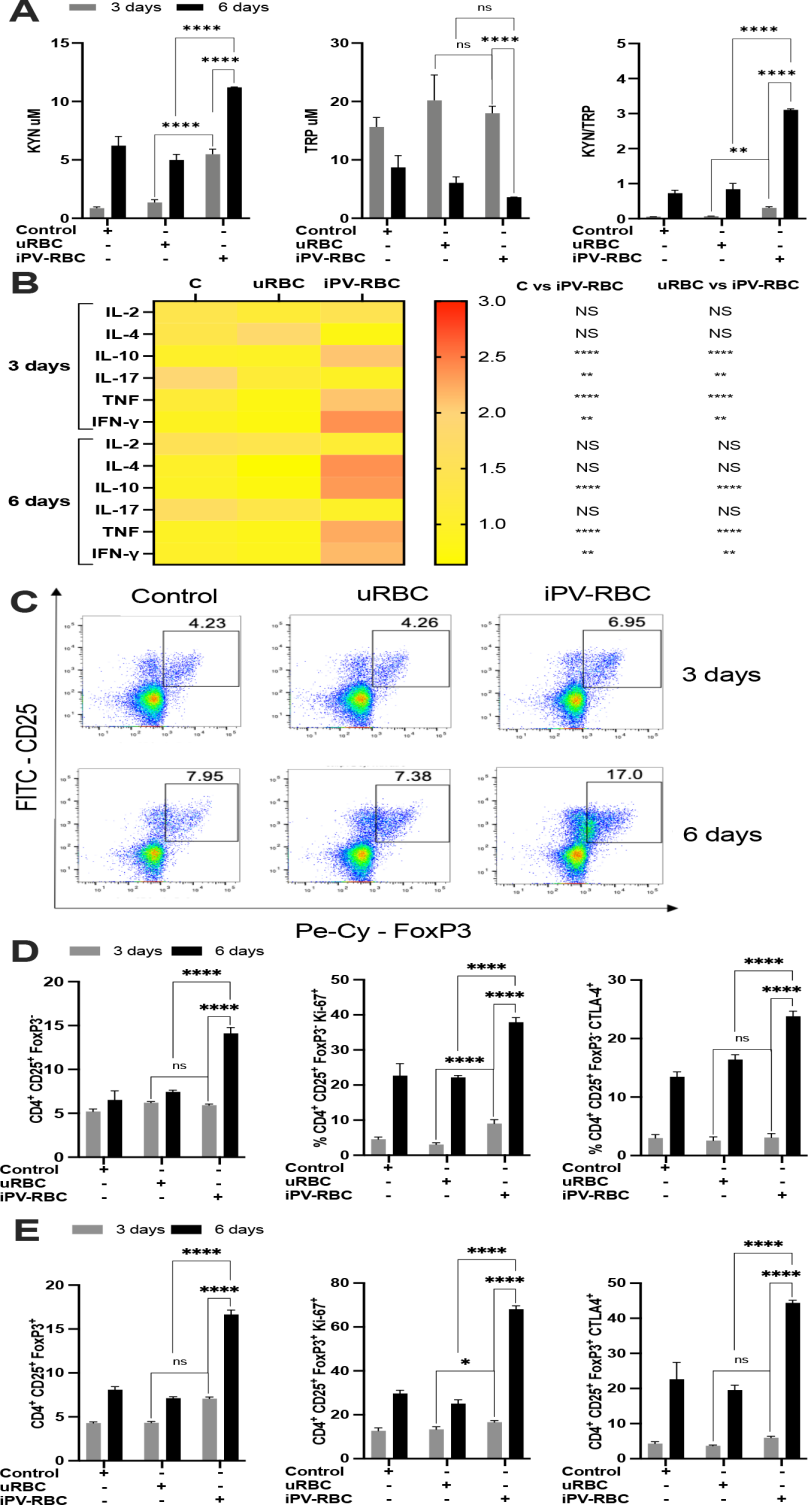
*Plasmodium vivax* stimulation induces and activates regulatory T cells (Treg cells). Healthy malaria-naïve donor PBMCs were cultured up to 6 days and treated with RPMI1640 with 10% growth medium as negative control, uninfected red blood cells (uRBCs) at 1:2 ratio, or iPV-RBC at 1:2 ratio. The cells were stained for T cell markers, and the percentage of CD4+CD25+FOXP3− and Treg (CD4+CD25+FOXP3+) cells was determined after 3 and 6 days of culture. **(A)** TRP and KYN and **(B)** cytokines were measured in cell supernatant after PBMC stimulation (**Supplementary Figure S2**). **(C)** Representative dot plot shows the CD4+ cells gated for CD25 and FoxP3 markers. Percentage of **(D)** CD4+CD25+FOXP3− and **(E)** CD4+CD25+FOXP3+ cell population post-stimulation. The data are shown as mean + SD of one representative donor PBMC stimulated in quadruplicate. The experiment was performed with five independent malaria-naïve donors. The heatmap is a visual representation of row-normalized cytokines, and Kruskal–Wallis with Dunn's multiple-comparisons test was used to compare the cytokines in **(B)**. Two-way ANOVA with Tukey's multiple-comparisons test was used for **(A, D, E)**. NS, not significant. *p < 0.05, **p < 0.01, and ****p < 0.0001.

Next, we evaluated the effect of *in vitro* stimulation of PBMCs with iPV-RBCs on the CD4+ T cell compartment. Vivax stimulation increased the IDO enzyme activity as measured by the KYN/TRP ratio (**Figure 2A**) and increased the production of cytokines measured in cell culture supernatants, notably, IL-2, IL-4, IL-17, TNF, and IFN-γ (**Figure 2B**; **Supplementary Figure 2**). We also observed signs of T cell proliferation and activation, evidenced by an increase in CD4+Ki67+ and CD4+CTLA4+ T cells after iPV-RBC when compared to uRBC stimulated PBMCs (**Supplementary Figure S3A**). *P. vivax* extract stimulation significantly increased the frequency, proliferation, and activation of CD4+CD25+FoxP3- (**Figure 2D**) and CD4+CD25+FoxP3+ Treg cell (**Figures 2C, E**).

**Figure 3:**
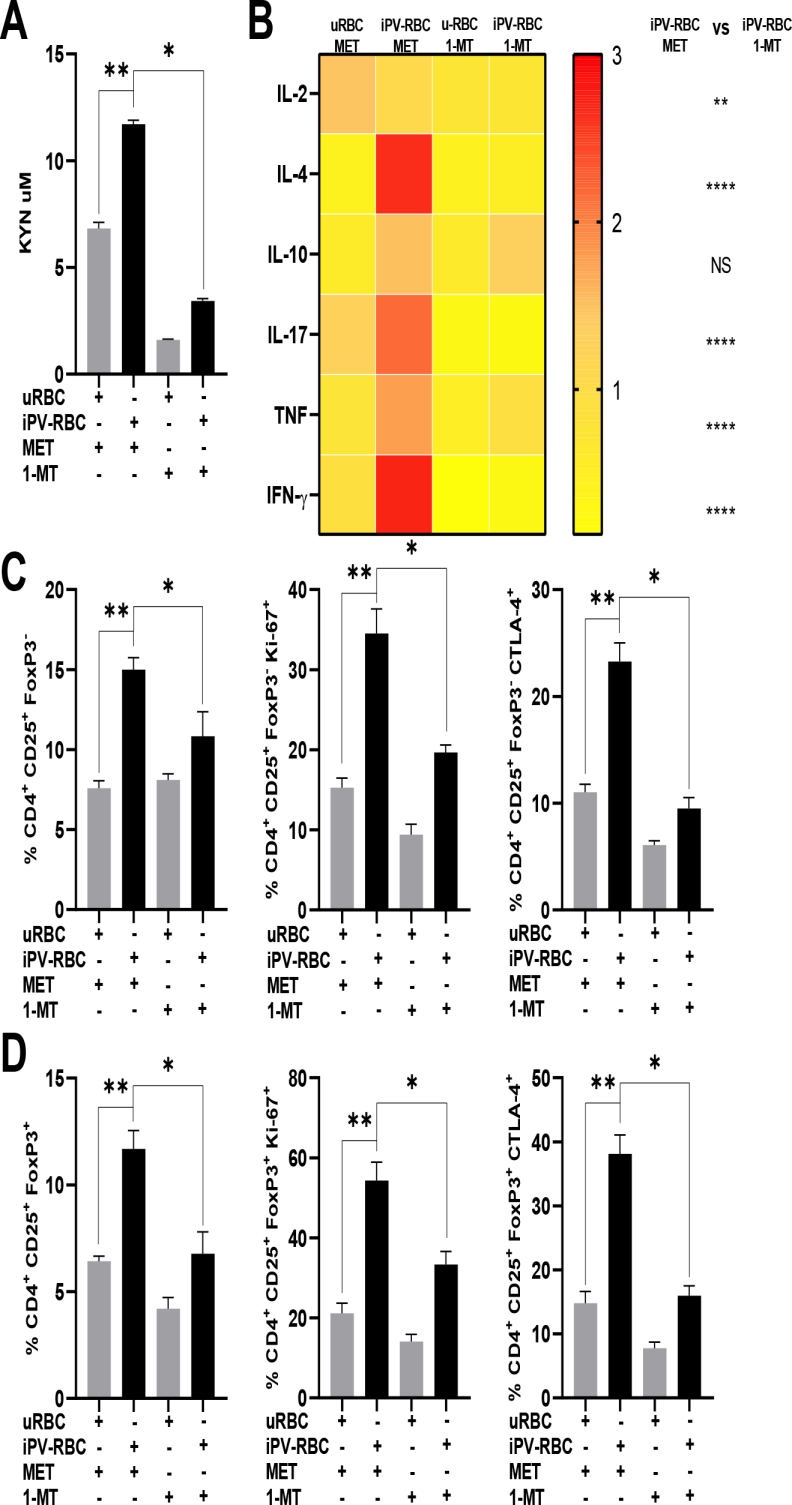
Inhibition of IDO enzyme decreases kynurenine and abrogates the induction of Treg cells. Healthy malaria-naïve donor PBMCs were cultured for up to 6 days and stimulated with uRBCs or iPV-RBC at 1:2 ratio in the presence of methanol (MET, vehicle) or 1-methyl tryptophan (1-MT), a potent and specific inhibitor of indoleamine 2,3-dioxygenase (IDO) enzymatic activity. **(A)** The KYN levels were quantified by HPLC, and **(B)** cytokines were measured by bead assay in cell supernatant after PBMC stimulation (**Supplementary Figure S4**). The heatmap was plotted using the means for each cytokine. The percentage of **(C)** CD4+CD25+FOXP3− and **(D)** CD4+CD25+FOXP3+ cell population post-stimulation, respectively, is described. The data are shown as mean + SD of one representative donor PBMC stimulated in quadruplicate. The experiment was performed with five independent malaria-naïve donors. Kruskal–Wallis with Dunn's multiple-comparisons test was used for **(A, C, D)**. The heatmap is a visual representation of row-normalized cytokines. Mann–Whitney test was used to compare the two groups in **(B)** on day 6. NS, not significant. *p < 0.05, **p < 0.01, and ****p < 0.0001.

To further characterize the role of IDO enzyme and its influence on the activation of T cells, we added to the cell culture a competitive inhibitor of the IDO enzyme, 1-methyl-D-tryptophan (1-MT). Inhibiting IDO significantly reduced the level of KYN metabolite in the cell culture supernatant of iPV-RBC stimulated cells compared to the control group (**Figure 3A**). IDO enzyme inhibition also significantly reduced the levels of cytokines IL-2, IL-4, TNF, and IFN-γ in the cell culture supernatant (**Figure 3B**, **Supplementary Figure 4**). We likewise observed a reduction in the frequencies of CD4+CD25+FoxP3- and Tregs by inhibiting IDO, which was accompanied by a reduction in Ki-67 and CTLA4 expression by these cells at day 6 post-stimulation (**Figures 3C, D**; **Supplementary Figure 5**).

**Figure 4:**
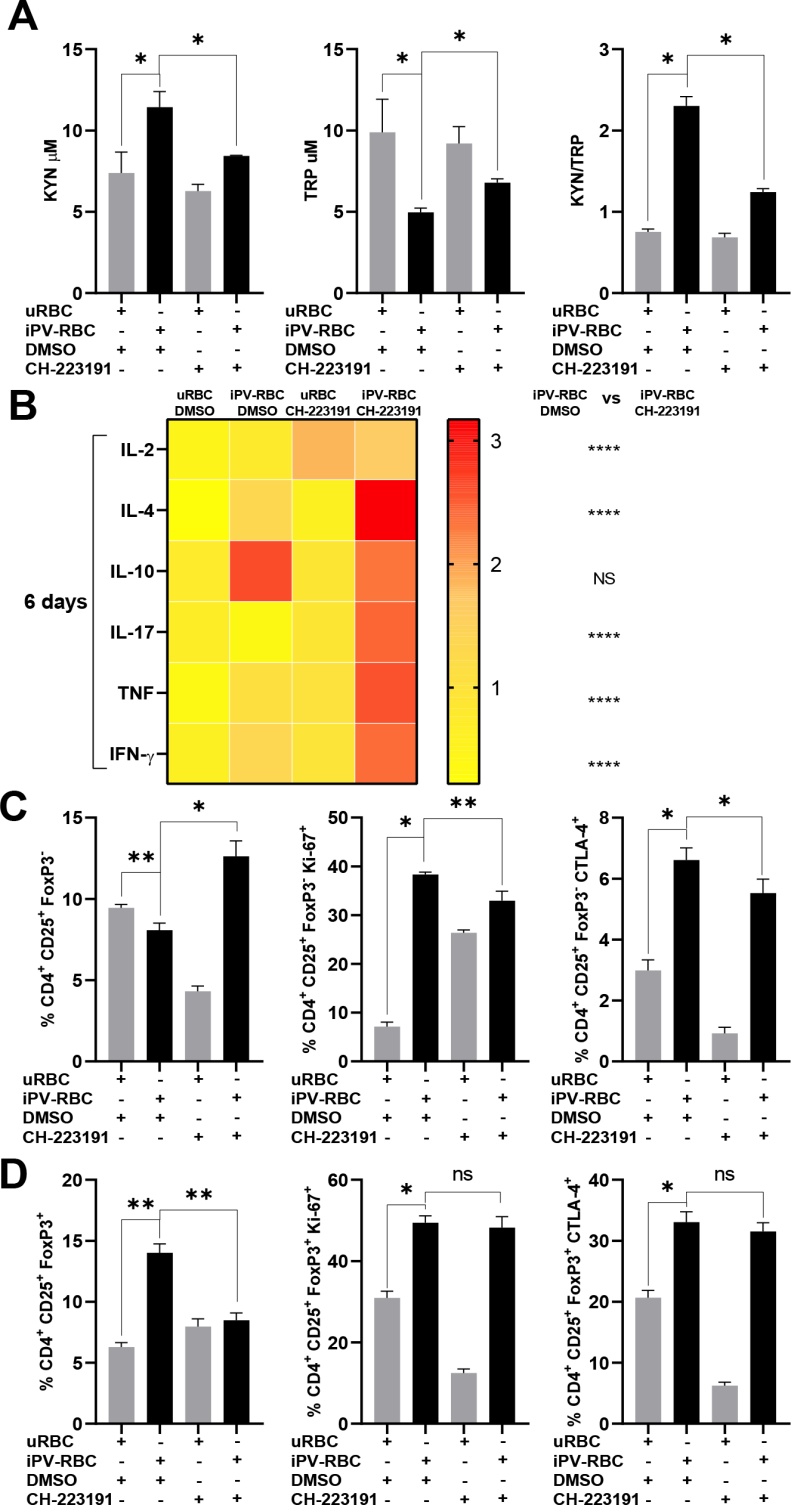
Inhibition of aryl hydrocarbon receptor (AhR) abrogates the induction of Treg cells. Healthy malaria-naïve donor PBMCs were cultured for up to 6 days and stimulated with uninfected red blood cells (uRBCs) or iPV-RBC at 1:2 ratio in the presence of DMSO (vehicle) or CH-223191, a potent and specific aryl hydrocarbon receptor (AhR) antagonist. **(A)** TRP and KYN and **(B)** cytokines were measured in cell supernatant after PBMC stimulation. The heatmap was plotted using the means for each cytokine (**Supplementary Figure S6**). The percentage of **(C)** CD4+CD25+FOXP3− and **(D)** CD4+CD25+FOXP3+ cell population post-stimulation, respectively, is described. The data are shown as mean + SD of one representative donor PBMC stimulated in quadruplicate. The experiment was performed with four independent malaria-naïve donors. Kruskal–Wallis with Dunn's multiple-comparisons test was used for **(A, C, D)**. The heatmap is a visual representation of row-normalized cytokines. Mann–Whitney test was used to compare differences in groups for **(B)** on day 6. NS, not significant. *p < 0.05, **p < 0.01, and ****p < 0.0001.

We next tested the hypothesis that iPV-RBC-induced increase of KYN mediates the expansion of regulatory T cells via the AhR using a AhR antagonist CH-223191. Inhibition of AhR reduced the KYN metabolite and KYN/TRP ratio in the culture supernatant after iPV-RBC stimulation (**Figure 4A**). On the other hand, AhR inhibition significantly increased the expression of several pro-inflammatory cytokines IL-2, IL-4, IL-17, TNF, and IFN-γ at day 6 after iPV-RBC stimulation (**Figure 4B**, **Supplementary Figure 6**). An increase in CD4+CD25+FoxP3- T cells by inhibiting AhR was accompanied by a reduction in proliferation (Ki-67) at day 6 and a decrease in CTLA-4 and CD45-RA (**Figure 4C**, **Supplementary Figure 7**). Interestingly, the frequency of Treg cells was reduced by inhibiting AhR, although there were no differences in the Ki-67 and CTLA-4 expression on Tregs (**Figure 4D**). In summary, innate immune activation via MyD88 induces the production of KYN metabolite, which binds AhR to induce Tregs.

### Demographic, hematological, and plasma cytokine characteristics of *P. vivax*-infected patients

Symptomatic individuals diagnosed at a malaria clinic were recruited to understand the relation between plasma kynurenines and Tregs. **Table 1** shows a summary of the characteristics of the study participants. Healthy endemic individuals without symptoms and fever were recruited as controls (*n* = 11) along with acute Pv-infected patients (*n* = 23). There was a predominance of men in both study groups, and the mean age of the study participants was 38 years. The median parasitic load estimated by thick blood smear was 3,083/mm^3^ (IQR, 533-2556) in the *P. vivax*-infected patients. Individuals with previous malaria infections had roughly twice the quantity of parasites per cubic millimeters compared to the first-time malaria patients. We observed that malaria infection induced significant hematological changes that included reduced peripheral leukocytes and platelets (**Table 1**). IL-6, IL-10, and IFN-γ were significantly elevated among acutely infected patients compared to the controls (**Table 2**). Interestingly, IFN-γ was significantly higher among individuals with first malaria infection compared to individuals with a previous malaria infection (**Table 2**). In contrast, IL-10 and IL-6 were elevated in the group of multiple infections when compared to individuals with their first malaria infection (**Table 2**).

**Table 1:** Patient clinical and demographic details.

Demographic Characteristics	Endemic Control	*P. vivax* (n=23)			*p value* ^AB^	*p value* ^AC^	*p value* ^AD^	*p value* ^CD^
	(n=11)^A^	Total (n=23)^B^	Episodes					
			1 (n=14)^C^	>1 (n=9)^D^				
Gender, Female (%)	2 (18,2%)	7 (30,43%)	5 (35,7%)	2 (22,2%)				
Age, mean (Years ± SD)	38.8 ± 12.2	38,6 ± 12.2	38.8 ± 12.2	38.9 ± 12.2				
Parasitemia, median (Parasites/mm3. IQR)		3083 (533 - 2556)	2432 (598.8 - 2240)	4096 (937,9 – 2814)				
Haematological parameters (mean ± SD)								
Haematocrit (%)	43.3 ± 3.3	43.6 ± 5.5	41.2 ± 3.1	42.2 ± 8.2	>0.9999	0.5810	>0.9999	>0.9999
Haemoglobin (g/dL)	13.3 ± 1.2	13.2 ± 1.8	13.2 ± 1.1	13.2 ± 2.6	>0.9999	>0.9999	>0.9999	>0.9999
White blood cells (x 10^3^/µL)	6.1 ± 1.4	4.5 ± 1.3	3.9 ± 1.1	5.2 ± 1.2	**0.0257**	**0.0014**	0.9073	0.0798
Red blood cells (x 10^6^/µL)	4.8 ± 4.9	4.8 ± 6.5	4.7 ± 3.6	4.8 ± 9.6	>0.9999	>0.9999	>0.9999	>0.9999
Mean corpuscular volume (fL)	89.3 ± 4.5	85.7 ± 2.6	85.8 ± 2.6	90.8 ± 5.7	**0.0375**	**0.0228**	>0.9999	**0.0322**
Mean corpuscular haemoglobin (pg)	27.6 ± 1.4	28.4 ± 2.2	28.2 ± 2.4	28.9 ± 1.9	0.7062	0.9390	0.2369	>0.9999
Mean corpuscular haemoglobin concentration (g/dL)	31.1 ± 0.9	32.1 ± 1.8	32.2 ± 1.6	31.8 ± 0.8	0.0691	**0.0449**	0.2465	>0.9999
Mean platelet volume (fL)	9.3 ± 0.9	10.6 ± 1.4	10.9 ± 1.4	9.9 ± 1.1	0.0724	**0.0162**	0.8766	0.6997
Platelet (x 10^3^/µL)	229 ± 48.6	96 ± 4.5	91 ± 41.1	105 ± 50.8	**<0.0001**	**<0.0001**	**0.0029**	>0.9999

1: First malaria; >1: malaria episodes; Statistically significant p-values are shown in bold.

**Table 2:** Blood cytokine levels in *P. vivax*-infected patients.

Cytokines pg/mL (mean ± SD)	Endemic control	Total (n = 23)^B^	*P. vivax*		*p value^AB^*	*p value^AC^*	*p value^AD^*	*p value^CD^*
			Episodes					
	(n = 11)^A^		1 (n = 14)^C^	>1 (n = 9)^D^				
IL-2	0.0 ± 0.0	0.1 ± 0.2	0.2 ± 0.4	0.1 ± 0.2	0.0694	0.7813	0.3991	>0.9999
IL-4	0.1 ± 0.2	0.8 ± 1.1	0.8 ± 1.2	0.8 ± 1.1	0.0255	0.1416	0.1761	>0.9999
IL-6	0.4 ± 0.3	126.9 ± 444.4	22.2 ± 27.9	289.7 ± 702.1	<0.0001	0.0004	0.0007	>0.9999
IL-10	0.1 ± 0.1	118.7 ± 286.3	33.6 ± 23.7	251.3 ± 438.3	<0.0001	0.0020	<0.0001	0.9871
IL-17	51.5 ± 69.3	17.9 ± 24.1	18.5 ± 21.9	16.9 ± 28.5	0.1433	>0.9999	0.2978	>0.9999
TNF	0.0 ± 0.0	0.48 ± 2.1	0.0 ± 0.0	1.2 ± 3.3	0.5349	>0.9999	0.3565	0.3042
IFN-*γ*	0.0 ± 0.0	91.5 ± 264.2	187.9 ± 389.0	25.1 ± 30.7	0.0002	0.0022	0.0724	0.0173

1, first malaria; >1, malaria episodes; Statistically significant p-values are shown in bold.

### Tregs cells increase in acute *P. vivax* malaria patients and positively correlate with tryptophan catabolism

**Figure 5:**
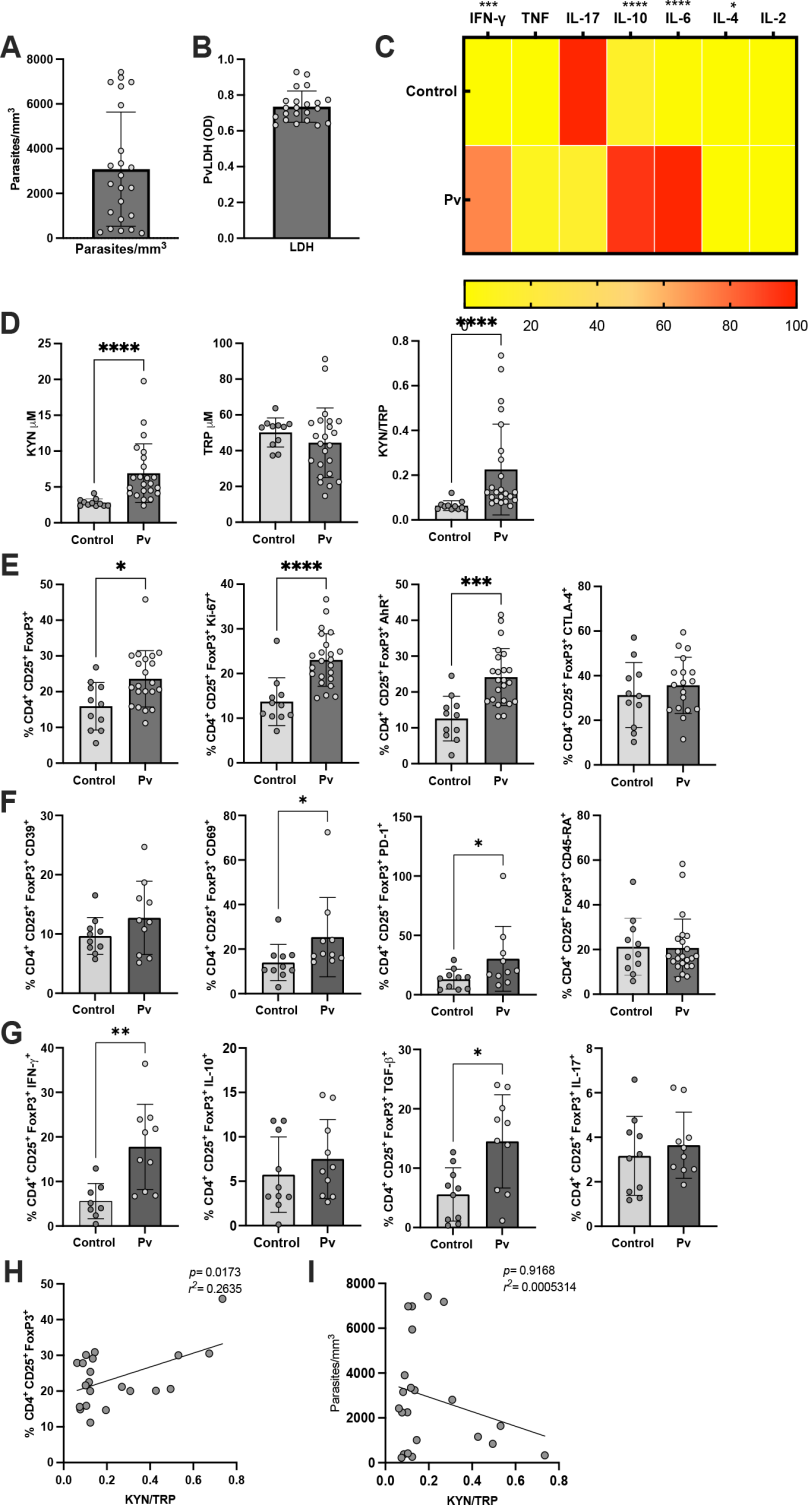
*Plasmodium vivax* infection *in vivo* triggers an increase in kynurenine metabolite and regulatory T cells. Endemic healthy controls (*n* = 11) and symptomatic *P. vivax* infection (*n* = 23) confirmed by light microscopy were used in this study. **(A)** Blood parasitemia levels were estimated by blood smear, and **(B)** plasma *P. vivax* LDH (PvLDH) was determined by sandwich ELISA. Plasma samples were used to measure **(C)** cytokines by bead assay and **(D)** KYN and TRP levels by HPLC. **(E–G)** CD4+CD25+FoxP3+ lymphocyte population and expressed markers and intracellular cytokines. Correlation between the KYN/TRP ratio and **(H)** CD4+CD25+FoxP3+ cells. **(I)** Parasite levels. The data are shown as mean ± SD. Mann–Whitney test was used to compare differences in groups for **(C–G)**. Pearson’s correlation was performed for data in **(H, I)**. *p < 0.05, **p < 0.01, ***p < 0.001, and ****p < 0.0001.

Blood parasitemia was calculated by measuring RBC infected with *P. vivax* parasites (**Figure 5A**), and *P. vivax* LDH was measured in plasma samples (**Figure 5B**). Overall, we observed an increase in proinflammatory cytokines during the blood-stage infection (**Figure 5C**). A significant increase in both plasma KYN metabolite and KYN/TRP ratio was observed among acute patients before starting the anti-malaria treatment (**Figure 5D**).

Next, we investigated Treg subsets and markers among healthy endemic controls and *P. vivax*-infected patients. Peripheral blood mononuclear cells (PBMCs) were analyzed directly without stimulation (*ex vivo*); we observed no difference in the percentage CD4+ and CD4+CD25+FoxP3- cell subsets between the study groups (**Supplementary Figures S8A**, **S9A**). On the other hand, a significant increase of Tregs cells was observed after acute malaria infection (**Figure 5E**). Additionally, we observed an increase in the expression of Ki-67, a proliferation marker, on CD4+, CD4+CD25+FoxP3- and CD4+CD25+FoxP3+ T lymphocytes in acute disease (**Supplementary Figures S8**). An increase in intracellular AhR-expressing lymphocyte subsets was also observed in acute malaria patients (**Figure 5E**). Inhibitory molecule PD-1 expression was higher on Tregs in acute symptomatic patients. CD39+Tregs with regulatory effector/memory phenotype were similar among healthy and acutely infected patients. Here we observed an overall increase in CD69+Tregs (**Figure 5F**). Additionally, no difference between control and patients on the expression of CD45-RA, a naïve T cell marker, was observed in the Tregs subsets (**Figure 5F**). In further analyzing Tregs, we observed a significant increase in intracellular IFN-γ and TGF-β Tregs after *P. vivax* infection. We did not observe a significant increase in IL-10 and IL-17 expression Tregs (**Figure 5G**). A significant positive correlation between classical regulatory CD4+CD25+FoxP3+ T cells and the KYN/TRP ratio and a negative correlation with the KYN/TRP ratio were observed in acute malaria infections (**Figures 5H, I**, respectively).

### Higher malaria episodes associated with lower Tregs

**Figure 6:**
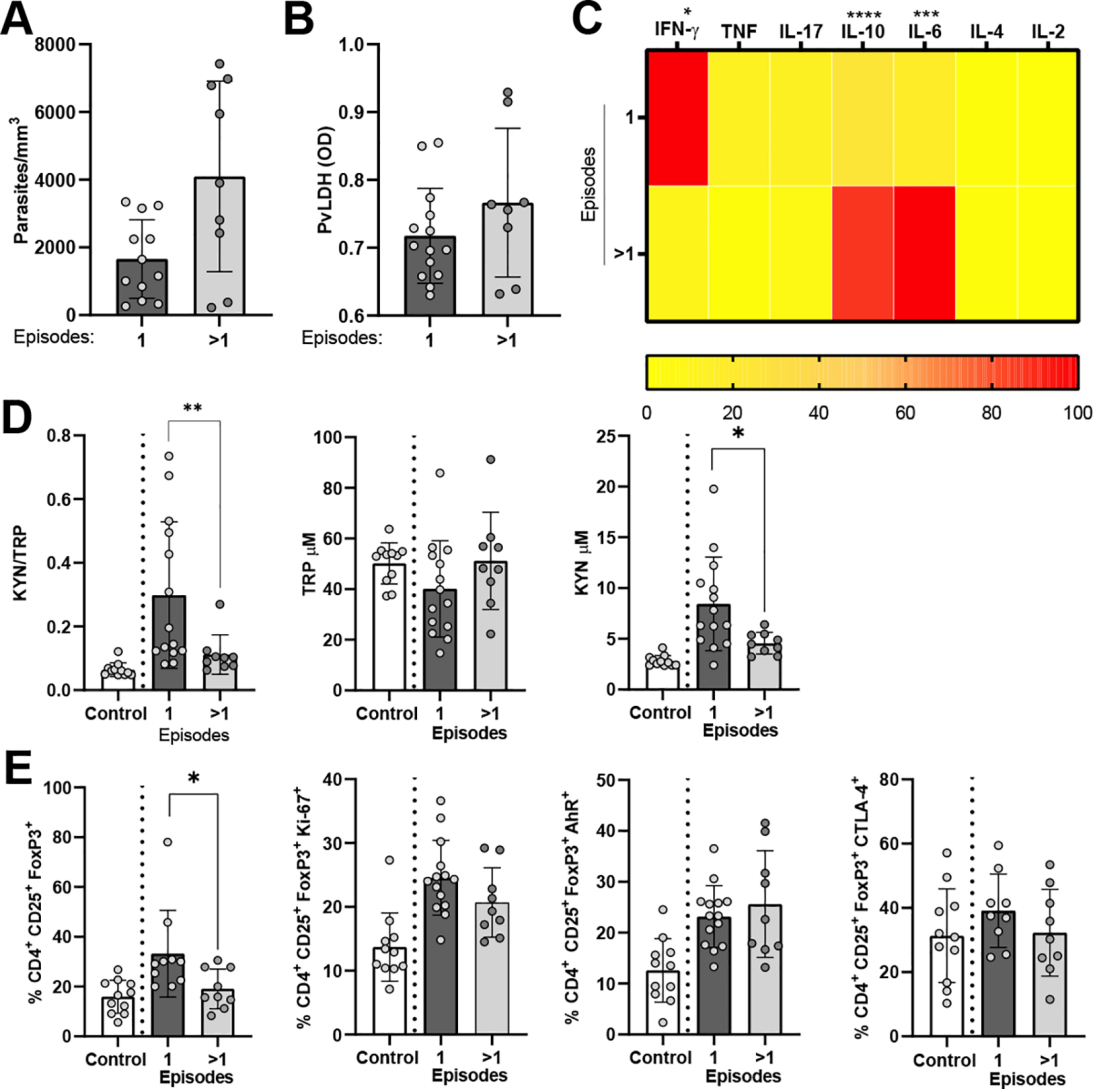
Kynurenine metabolite and regulatory T cells decline in individuals with *P. vivax* reinfection. *P. vivax*-infected patients with first infection (one episode, *n* = 14) or reinfection (>1 episode, *n* = 9) were compared. **(A)** Blood parasitemia levels were estimated by blood smear, and **(B)** plasma *P. vivax* LDH (PvLDH) was determined by sandwich ELISA. Plasma samples were used to measure **(C)** cytokines by bead assay and **(D)** KYN and TRP levels by HPLC. **(E)** CD4+CD25+FoxP3+ lymphocyte population and expressed markers. The data are shown as mean ± SD. The heatmap is a visual representation of row-normalized cytokines. Mann–Whitney test was used to compare differences in groups for **(A–E)**. *p < 0.05, **p < 0.01, ***p < 0.001, and ****p < 0.0001.

Next, we analyzed whether a previous malaria infection played a role in the modulation of immune response during malaria acute infection. Firstly, we observed a higher parasite load in individuals with a previous malaria episode (>1) compared to individuals with a first malaria infection (**Figure 6A**). On the contrary, we did not observe a difference in plasma LDH levels among the two study groups (**Figure 6B**). More importantly, we observed significantly higher IL-6 and IL-10 levels among individuals with previous malaria. On the other hand, the plasma IFN-γ levels were significantly elevated in individuals with first-time malaria compared to those with a reinfection (**Figure 6C**). The plasma levels of KYN metabolite and IDO enzyme activity were also significantly elevated in first-time malaria-infected patients (**Figure 6D**).

The CD4+ cell frequencies were similar between the two exposure groups; however, the frequency of proliferating Ki67+CD4 was higher among individuals with a first-time malaria (**Supplementary Figure S10A**). Individuals with a previous malaria infection and individuals with a first episode had a similar frequency of CD4+C25+FoxP3-. However, reinfected individuals expressed lower AhR and CTLA4 (**Supplementary Figure S10B**). Surprisingly, we observed a significantly reduced frequency of Tregs among patients with a previous malaria exposure compared to individuals with a first-time malaria. Although not significant, we observed a reduced frequency of CD4+C25+FoxP3+ expressing Ki-67 and CTLA-4 among patients with a previous malaria exposure (**Figure 6E**).

## Discussion

Physiopathology of acute infections is driven by rapid changes in the host metabolism and immune response. Expansion of T regulatory cells during blood-stage infection has been described in human and mice *Plasmodium* infection (8). However, the mechanisms that lead to its increase and role of host metabolism are not completely understood. In this study, we demonstrate that innate activation via MyD88 adaptor protein increases tryptophan catabolism to kynurenine by upregulating the expression of the IDO enzyme. Overall, KYN metabolite increased the frequencies of Treg and CTLA4 expression on CD4 and Treg cells by direct activation of AhR. Acute infection with *P. vivax* induced the expansion of CD4+CD25+FoxP3+ regulatory T cells (Tregs), where a positive correlation between Tregs and kynurenine metabolite was observed. Besides that, we also observed decreased levels of Treg cells and kynurenine metabolite levels in patients with a previous malaria infection. These results provide new insight into the critical mechanisms of immunosuppression associated with blood-stage malaria that impede the development of potent long-term adaptive immunity to reinfections.

*In vitro* stimulation of PBMCs with freeze–thaw lysate of *P. vivax*-infected erythrocytes (iPV-RBC) simulated the activation of pattern recognition receptors (PRRs) by parasite ligands. Here blocking MyD88 upon vivax stimulation reduced the KYN and IDO1 enzyme expression. Previously, the activation of MyD88 via TLR was shown to trigger an innate immune response (12). Malaria is characterized by fever and systemic inflammation during blood-stage infection. The innate immune system responds to parasites and parasite components by inducing pro-inflammatory cytokines and chemokines such as IL-1β, IL-6, IL-8, IL-12(p70), IFN-ɣ, and TNF. Some of these cytokines have also been demonstrated to upregulate IDO enzyme expression and increase tryptophan breakdown. However, further studies aiming to determine the specific PAMPs and PRRs important in the process of increasing tolerogenic metabolite kynurenine will be important for an overall understanding of the physiopathology of malaria.

Additionally, stimulation of PBMCs with the extract of *P. falciparum*-infected erythrocytes isolated from patients increased proinflammatory cytokines and induced Tregs (13, 14). The extract of falciparum-infected erythrocytes was shown to induce memory T cells in converting into iTreg cells (13). In other systems, KYN binding to AhR was shown to convert naive T cells into Tregs (7). Here we observed a decrease in CD45RA-positive cells upon *P. vivax* antigen stimulation. However, CD45RA-positive cell data was not consistent when the IDO enzyme or AhR was blocked. Hence, further experiments are needed to understand the role of naïve and memory cell compartment in the induction of Treg cells upon vivax stimulation. Nevertheless, IDO and AhR inhibitors in PBMC stimulated with *P. vivax* antigens efficiently reduced the KYN/TRP ratio and Treg cell frequencies. The inhibition of IDO enzyme also reduced the IFN-γ levels, whereas the inhibition of AhR elevated the IFN-γ levels. IFN-γ has been shown to play an important role in IDO enzyme induction and tryptophan catabolism (10, 15). Here we observed that KYN metabolite was essential for inducing Tregs. We believe that in addition to PRR activation by parasite PAMPS, AhR might also positively regulate IDO enzyme in myeloid cells (16, 17). Thus, blocking of AhR in *P. vivax*-stimulated cells reduced the KYN/TRP ratio even in the presence of high levels of IFN-γ. Furthermore, *P. falciparum*-infected erythrocytes were shown to induce Tregs *in vitro* independent of direct TCR stimulation but was dependent on IL-10 and TGF-β (14). In our study, we observed an increase in IL-10 post-stimulation with *P. vivax* extract; however, IDO or AhR inhibition also reduced the IL-10 levels. This implies that IL-10 might be indirectly regulated by KYN production; previously, AhR was shown to promote IL-10 expression in myeloid cells through the Src-STAT3 pathway (18). Generally, blocking the IDO enzyme *in vivo* and experiments with AhR knockout mice demonstrate an essential role of these molecules in reducing parasitemia (19–22). Although IDO and AhR play a significant role in the pathogenesis of the disease and its complications, the bulk of the data is either from *in vitro* cell studies or mouse models. More human data is likewise needed to establish the relevance of these findings. A further analysis of endogenous AhR ligands could improve our understanding of its role in the clinical spectrum of malaria (21, 23, 24).

Additionally, the observed simultaneous increase in IL-10 and pro-inflammatory cytokines in *P. vivax*-infected patients could be a result of the host’s immunoregulatory response to balance inflammation and prevent excessive immune-mediated tissue damage. *P. vivax* infection also induces a strong pro-inflammatory response, characterized by elevated levels of cytokines such as IFN-γ, TNF-α, and IL-6. In response, IL-10 is upregulated as a compensatory mechanism to limit excessive inflammation and mitigate potential immunopathology. Moreover, *P. vivax* infection has been associated with the increased activation of Tregs and monocytes, which are major sources of IL-10. This regulatory pathway is crucial in maintaining immune homeostasis and preventing hyperinflammation. Overall, we understand that there are mechanisms which not only depend on the number of malaria infections but also on the parasite load. Further studies are necessary to understand these responses, which are not the scope of this paper.

The elevated plasma KYN/TRP ratio and Tregs among acute *P. vivax*-infected patients observed in this study corroborates with recent controlled human *P. vivax* infection (25) and results from other studies (11, 26–28). Additionally, while we observed trends in certain immune markers, such as IL-17 and IFN-γ, the lack of strong statistical correlations may be due to the small sample size, which may limit the generalizability of our findings. A key limitation of our study is the unexpectedly high IL-17 expression observed in the endemic control group, which may reflect underlying environmental or immunological factors not fully accounted for. Additionally, while we noted similar IL-17 expression patterns in *P. vivax*-infected patients post-treatment, we were unable to confirm the mechanistic basis for this elevation (data not shown). In *P. vivax* infection, regulatory T cells (Tregs) and CD4+ cells expand and express inhibitory molecules such as CTLA-4 and PD-1, which play key roles in limiting immune responses (29). In this study, increased CTLA-4 expression on CD4+ cells and Tregs was observed following *in vitro* stimulation, but this was reduced when IDO or AhR was blocked. While CTLA-4+ Tregs were not significantly elevated in acute *P. vivax*-infected patients, there was a marked increase in PD-1+ Tregs. The lack of a significant elevation of CTLA-4+ Tregs in acute patients suggests the need for more detailed longitudinal studies to better understand the dynamic regulation of these inhibitory pathways. Additionally, the potential instability of FoxP3 expression may limit the interpretation of Treg function, and further studies are necessary to clarify the role of CD4+CD25+FoxP3- cells in immune regulation. Lastly, the cross-sectional nature of our data limits our ability to draw conclusions about causality.

We observed lower regulatory T cells and kynurenine metabolite in individuals with a previous malaria exposure. Previously, a decline in FoxP3+ regulatory cells was observed among children heavily exposed to *P. falciparum* (30). We observed higher parasite loads among individuals with a previous malaria infection compared with malaria-naïve individuals as previously described (10). Potent innate immune response is activated at a low blood parasitemia, similarly to what is observed in the experimental clinical setting (10, 25). However, we did not observe a positive correlation with parasite load as previously described in *P. vivax*-infected patients (26), but we observed an increase in AhR expressing Tregs in acute *P. vivax* infection. Overall, the adaptive immune response following *P. vivax* infection is often insufficient to confer long-term protection against reinfections. However, the underlying reasons for the observed higher parasitic load in previously infected patients remain unclear. We do not have definitive conclusions regarding this relationship and have reported it as an observation in our study. Several factors may contribute to this phenomenon, including immune tolerance or exhaustion due to repeated malaria exposure, which could reduce the efficiency of parasite control in subsequent infections. Additionally, variability in host immune responses, such as differences in cytokine regulation and effector cell activation, might influence parasite clearance rates. Moreover, because most vivax–malaria studies do not distinguish between first-time infections and previous exposures, it remains difficult to contextualize our findings within the broader literature. Understanding how repeated infections shape immune responses over time remains an open question, and future studies focusing on immune memory, RBC susceptibility, host metabolism, and host–parasite interactions in sequential malaria infections may provide further insights.

Here we did record the timing of a previous malaria exposure, but this information was based on individual memory and self-reports. We also did not observe any clear association between the time since the previous exposure and the level of parasitic load upon reinfection. During recruitment, we ruled out relapse as a cause of the observed reinfection. Currently, there are very few studies investigating the persistence and durability of T-cell responses specifically in *P. vivax* infection. However, both our study and others have observed recurrent infections occurring just a couple of months after the previous infection in the same individuals. This suggests that the immune response induced by *P. vivax* infection may be insufficient to provide long-lasting protection, leading to recurrent infections. Due to the limited number of PBMCs obtained from patients, we were unable to study the memory response and perform an in-depth analysis of CD4 T cell markers in this study. The primary objective of our study was to understand the mechanisms leading to the increase in Tregs in *P. vivax* infection. While examining memory T cells could provide valuable insights into the relationship between a prior malaria exposure and Treg expression, our data focus on the novel mechanisms of innate activation-induced tolerogenic phenotypes in *P. vivax* infection, particularly the role of tryptophan metabolism. We believe that our findings contribute new perspectives on how metabolic pathways influence immune regulation in *P. vivax* infection, and further studies with larger sample sizes and detailed T cell profiling will be needed to explore this relationship more thoroughly.

In this study, we demonstrate here that chronic malaria antigen exposure changes the host metabolic, cytokine, peripheral CD4+, and T regulatory cell immune response to acute infection. The observed negative correlation with parasite load and innate activation or tryptophan catabolism suggests that the stability and homeostasis of tolerogenic response are disturbed under highly inflammatory conditions. The implications of this pathogen-driven Treg loss for pathogen clearance, host–parasite equilibrium, and the development of clinical immunity in regions of intense malaria transmission require further investigation. In summary, the accelerated metabolism of TRP into KYN during the acute phase of *P. vivax* infection promotes an immunosuppressive environment with an induction of Tregs. However, additional studies are needed to understand not only the molecular mechanisms involved in innate immune activation, AhR–ligand interaction, and Treg induction but also its implication in impairing an efficient immune response.

## Data Availability

The original contributions presented in the study are included in the article/**Supplementary Material**. Further inquiries can be directed to the corresponding author.

## Supplementary material

Supplementary Figure 1 IDO1 and IDO2 gene expression is elevated upon *Plasmodium vivax*-infected erythrocytes (iPV-RBC) stimulation of PBMC. *In vitro* healthy malaria-naïve donor PBMCs were cultured for up to 3 and 6 days treated with RPMI1640 with 10% growth medium as negative control, uninfected red blood cells (uRBCs), or iPV-RBC (ratio: one PBMC is to two parasites; equivalent to 2 × 10^6^ parasites/mL). **(A)** IDO1 gene expression normalized to housekeeping gene and **(B)** percentage of increase in IDO1 expression compared to negative control. **(C)** IDO2 gene expression normalized to housekeeping gene and **(D)** percentage of increase in IDO2 expression compared to the negative control. The experiment was performed with five independent malaria-naïve donors. Two-way ANOVA with Tukey's multiple-comparisons test was used. ***p* < 0.01 and *****p* < 0.0001.

Supplementary Figure 2 Cytokine measurement after *Plasmodium vivax*-infected erythrocyte (iPV-RBC) stimulation of PBMC. *In vitro* healthy malaria-naïve donor PBMCs were cultured for up to 3 and 6 days treated with RPMI1640 with 10% growth medium as negative control, uninfected red blood cells (uRBCs), or iPV-RBC. Cytokines were measured by bead assay in cell supernatant after PBMC stimulation. Two-way ANOVA with Tukey's multiple-comparisons test was used. ***p* < 0.01 and *****p* < 0.0001.

Supplementary Figure 3 CD4+ cell proliferation and CTLA-4+ increase in *Plasmodium vivax*-infected erythrocyte (iPV-RBC) stimulation of PBMC. *In vitro* healthy malaria-naïve donor PBMCs were cultured for up to 3 and 6 days and treated with RPMI1640 with 10% growth medium as negative control, uninfected red blood cells (uRBCs), or iPV-RBC. CD4 T cell markers after vivax stimulation in the **(A)** absence or presence of **(B)** IDO and **(C)** AhR inhibitor. The data are shown as mean + SD of one representative donor PBMC stimulated in quadruplicate. The experiment was performed with five independent malaria-naïve donors. Two-way ANOVA with Tukey's multiple-comparisons test was used. NS, not significant. **p* < 0.05, ***p* < 0.01, ****p* < 0.001, and *****p* < 0.0001.

Supplementary Figure 4 Cytokine measurement after *Plasmodium vivax*-infected erythrocyte (iPV-RBC) stimulation of PBMC in the presence of IDO inhibitor. Healthy malaria-naïve donor PBMCs were cultured for up to 6 days and stimulated with uninfected red blood cells (uRBCs) or iPv-RBC at 1:2 ratio in the presence of methanol (MET, vehicle) or 1-methyl tryptophan (1-MT), a potent and specific inhibitor of indoleamine 2,3-dioxygenase (IDO) enzymatic activity. Cytokines were measured by bead assay in cell supernatant after PBMC stimulation. Two-way ANOVA with Tukey's multiple-comparisons test was used. ***p* < 0.01 and *****p* < 0.0001.

Supplementary Figure 5 IDO enzyme inhibition and induction of Tregs. Healthy malaria-naïve donor PBMCs were cultured for up to 6 days and stimulated with uninfected red blood cells (uRBCs) or iPv-RBC at 1:2 ratio in the presence of methanol (MET, vehicle) or 1-methyl tryptophan (1-MT), a potent and specific inhibitor of indoleamine 2,3-dioxygenase (IDO) enzymatic activity. **(A)** TRP and KYN and **(B)** cytokines were measured in cell supernatant after PBMC stimulation. The heatmap was plotted using the means for each cytokine. The percentage of **(C)** CD4+CD25+FOXP3− and **(D)** CD4+CD25+FOXP3+ cell population stimulation, respectively, is described. The data are shown as mean ± SD of one representative donor PBMC stimulated in quadruplicate. The experiment was performed with four independent malaria-naïve donors. Two-way ANOVA with Tukey's multiple-comparisons test was used for **Supplementary Figures S3A, C, D**. The heatmap is a visual representation of row-normalized cytokines, and statistical differences were observed on day 6. Mann–Whitney test was used to compare differences in groups for **Supplementary Figure S3B**. NS, not significant. **p* < 0.05, ***p* < 0.01, ****p* < 0.001, and *****p* < 0.0001.

Supplementary Figure 6 Cytokine measurement after *Plasmodium vivax*-infected erythrocyte (iPV-RBC) stimulation of PBMC in the presence of AhR inhibitor. Healthy malaria-naïve donor PBMCs were cultured for up to 6 days and stimulated with uninfected red blood cells (uRBCs) or iPv-RBC at 1:2 ratio in the presence of DMSO (vehicle) or CH-223191, a potent and specific aryl hydrocarbon receptor (AhR) antagonist. Cytokines were measured by bead assay in cell supernatant after PBMC stimulation Two-way ANOVA with Tukey's multiple-comparisons test was used. **p* < 0.05, ***p* < 0.01, ****p* < 0.001, and *****p* < 0.0001.

Supplementary Figure 7 Inhibition of aryl hydrocarbon receptor (AhR) abrogates the induction of Treg. Healthy malaria-naïve donor PBMCs were cultured for up to 6 days and stimulated with uninfected red blood cells (uRBCs) or iPv-RBC at 1:2 ratio in the presence of DMSO (vehicle) or CH-223191, a potent and specific aryl hydrocarbon receptor (AhR) antagonist. **(A)** TRP and KYN and **(B)** cytokines were measured in cell supernatant after PBMC stimulation at day 3 and 6. Statistical data is compared at day 6. Percentage of **(C)** CD4+CD25+FOXP3− and **(D)** CD4+CD25+FOXP3+ cell population post-stimulation is described. The data are shown as mean ± SD of one representative donor PBMC stimulated in quadruplicate. The experiment was performed with four independent malaria-naïve donors. Two-way ANOVA with Tukey's multiple-comparisons test was used for **Supplementary Figures S4A, C, D**. The heatmap is a visual representation of row-normalized cytokines, and statistical differences were observed on day 6, Mann–Whitney test was used to compare differences in groups for **Supplementary Figure S4B**. NS, not significant. **p* < 0.05, ***p* < 0.01, ****p* < 0.001, and *****p* < 0.0001.

Supplementary Figure 8 CD4 T cell surface and intracellular marker expressed in *P. vivax*-infected patients. Endemic healthy controls (*n* = 11) and symptomatic *P. vivax* infection (*n* = 23) confirmed by light microscopy were used in this study. **(A–C)** CD4+ lymphocyte population and expressed markers and intracellular cytokines. The data are shown as mean ± SD. Mann–Whitney test was used to compare differences in groups. **p* < 0.05 and ***p* < 0.01.

Supplementary Figure 9 CD4+CD25+FOXP3- T marker expressed in *P. vivax*-infected patients. Endemic healthy controls (*n* = 11) and symptomatic *P. vivax* infection (*n* = 23) confirmed by light microscopy were used in this study. **(A–C)** CD4+CD25+FOXP3+ lymphocyte population and expressed markers and intracellular cytokines. The data are shown as mean ± SD. Mann-Whitney test was used to compare differences in groups. **p* < 0.05, ***p* < 0.01, and ****p* < 0.001.

Supplementary Figure 10 CD4+ and CD4+CD25+FoxP3- in individuals with *P. vivax* reinfection. *P. vivax*-infected patients with first infection (one episode, *n* = 14) or reinfection (>1 episode, *n* = 9) were compared. **(A)** CD4+ and **(B)** CD4+CD25+FoxP3- lymphocyte population and expressed markers. The data are shown as mean ± SD. Mann–Whitney test was used to compare differences in groups. **p* < 0.05.

Supplementary Figure 11 Flow cytometry gating strategy.

Supplementary Figure 12 FMO analysis (also refer to **Supplementary Table S3**).
