# Supplementary material for: IDO1-AhR axis increases T regulatory cells in Plasmodium vivax malaria infection
Source: Front Immunol. 2025 Jul 14;16:1474447. doi: 10.3389/fimmu.2025.1474447 (PMC12301311; doi:10.3389/fimmu.2025.1474447)
Supplement: Supplementary Figure 1 — IDO1 and IDO2 gene expression is elevated upon Plasmodium vivax-infected erythrocytes (iPV-RBC) stimulation of PBMC. In vitro healthy malaria-naïve donor PBMCs were cultured for up to 3 and 6 days treated with RPMI1640 with 10% growth medium as negative control, uninfected red blood cells (uRBCs), or iPV-RBC (ratio: one PBMC is to two parasites; equivalent to 2 × 10^6^ parasites/mL). (A) IDO1 gene expression normalized to housekeeping gene and (B) percentage of increase in IDO1 expression compared to negative control. (C) IDO2 gene expression normalized to housekeeping gene and (D) percentage of increase in IDO2 expression compared to the negative control. The experiment was performed with five independent malaria-naïve donors. Two-way ANOVA with Tukey's multiple-comparisons test was used. **p < 0.01 and ****p < 0.0001. [file DataSheet1.pdf]

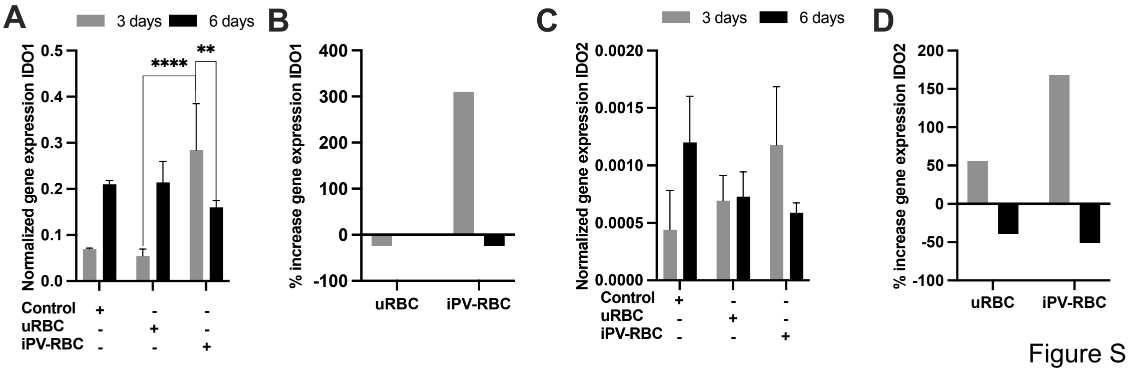
A
3 days
6 days
Normalized gene expression IDO1
0.5
0.4
0.3
0.2
0.1
0.0
****
**
Control + - -
uRBC - + -
iPV-RBC - - +
B
% increase gene expression IDO1
400
300
200
100
0
-100
uRBC
iPV-RBC
C
3 days
6 days
Normalized gene expression IDO2
0.0020
0.0015
0.0010
0.0005
0.0000
Control + - -
uRBC - + -
iPV-RBC - - +
D
% increase gene expression IDO2
200
150
100
50
0
-50
-100
uRBC
iPV-RBC

Figure S1

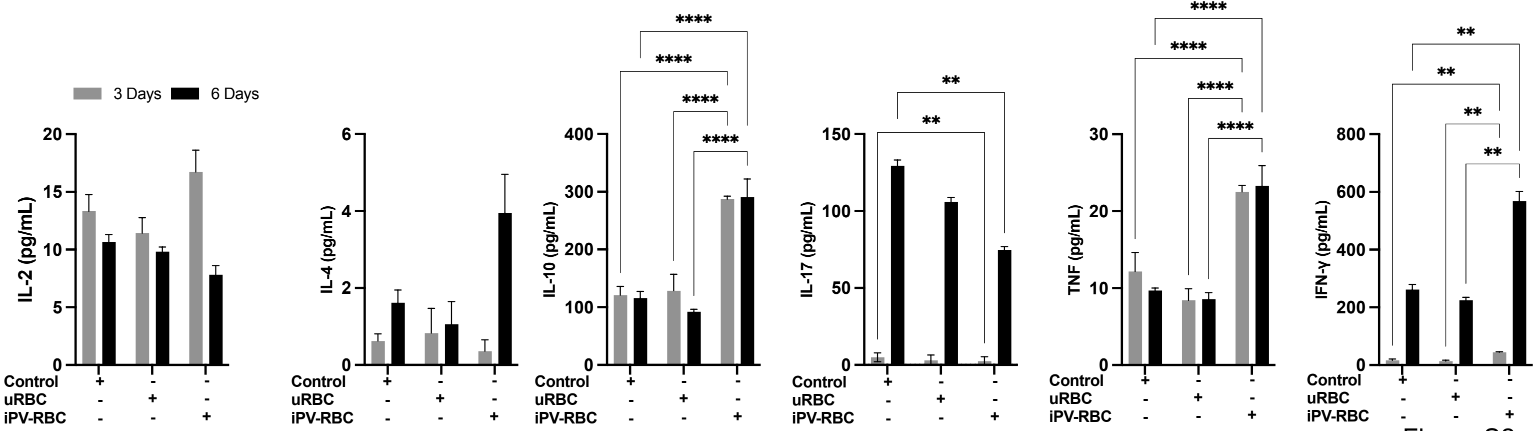
3 Days
6 Days
IL-2 (pg/mL)
IL-4 (pg/mL)
IL-10 (pg/mL)
IL-17 (pg/mL)
TNF (pg/mL)
IFN-γ (pg/mL)
****
**
Control
uRBC
iPV-RBC

Figure S2

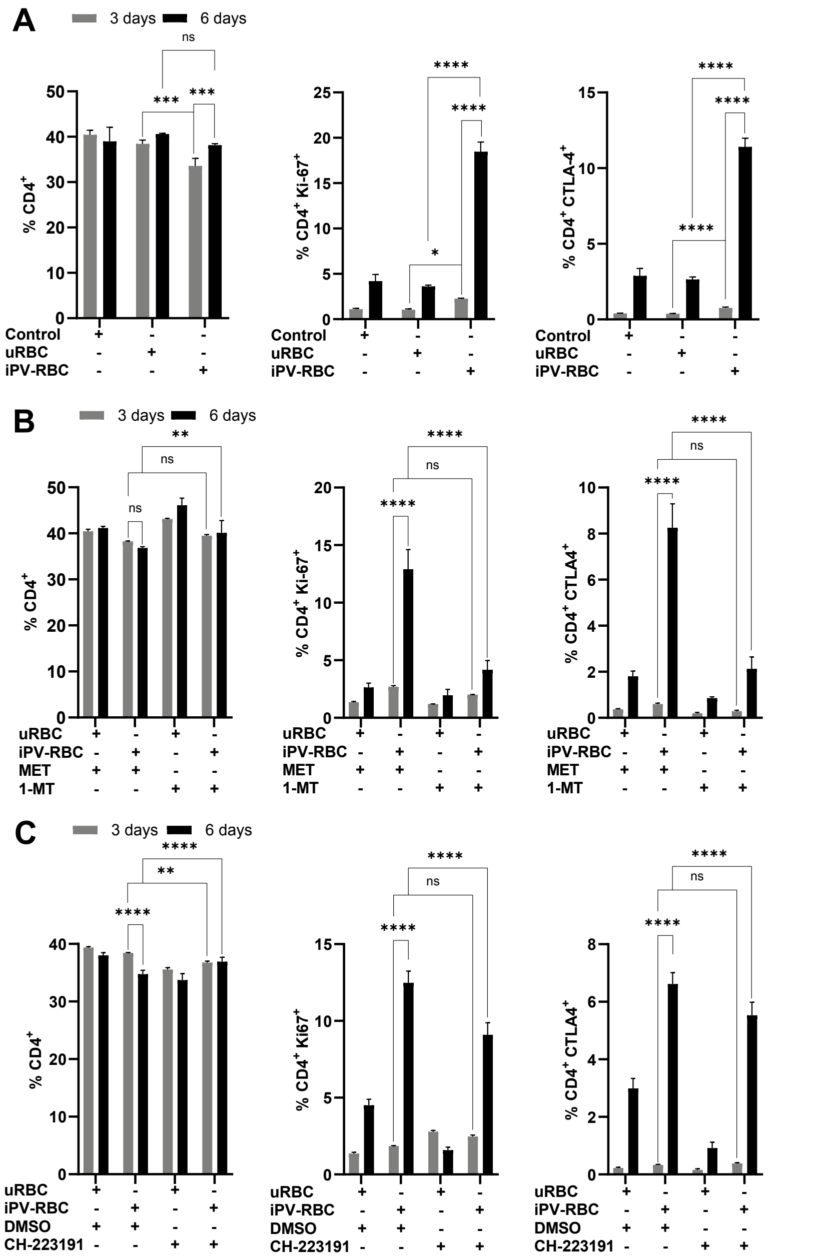
A
3 days
6 days
ns
***
***
****
****
*
****
****
****
% CD4+
% CD4+ Ki-67+
% CD4+ CTLA-4+
Control
uRBC
iPV-RBC
B
3 days
6 days
**
ns
ns
****
ns
****
****
ns
****
% CD4+
% CD4+ Ki-67+
% CD4+ CTLA4+
uRBC
iPV-RBC
MET
1-MT
C
3 days
6 days
****
**
****
****
ns
****
****
ns
****
% CD4+
% CD4+ Ki67+
% CD4+ CTLA4+
uRBC
iPV-RBC
DMSO
CH-223191

Figure S3

Figure S4

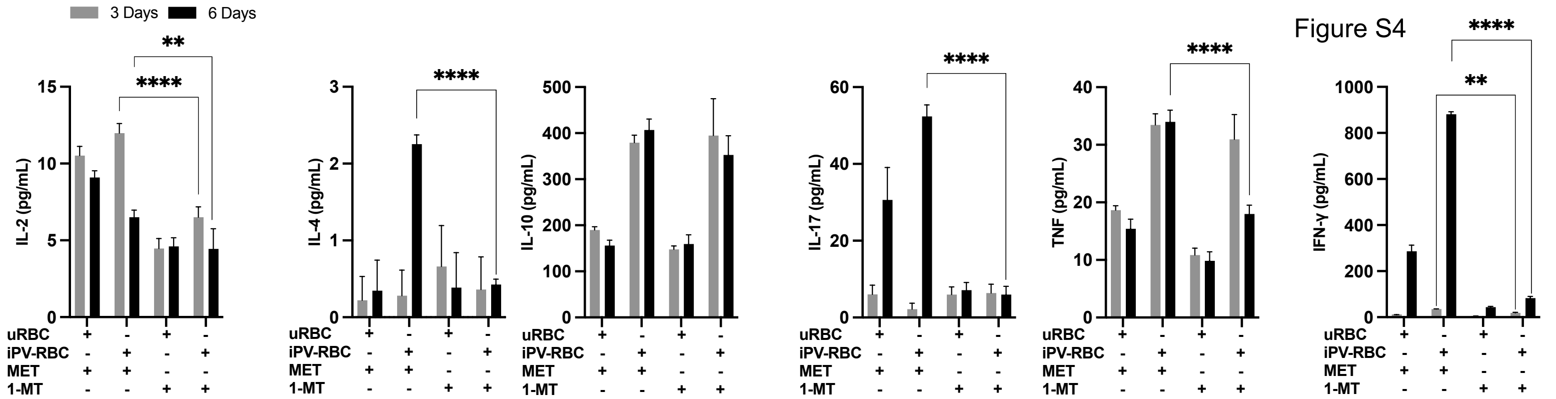

3 Days
6 Days
IL-2 (pg/mL)
IL-4 (pg/mL)
IL-10 (pg/mL)
IL-17 (pg/mL)
TNF (pg/mL)
IFN-γ (pg/mL)
**
****
uRBC
iPV-RBC
MET
1-MT

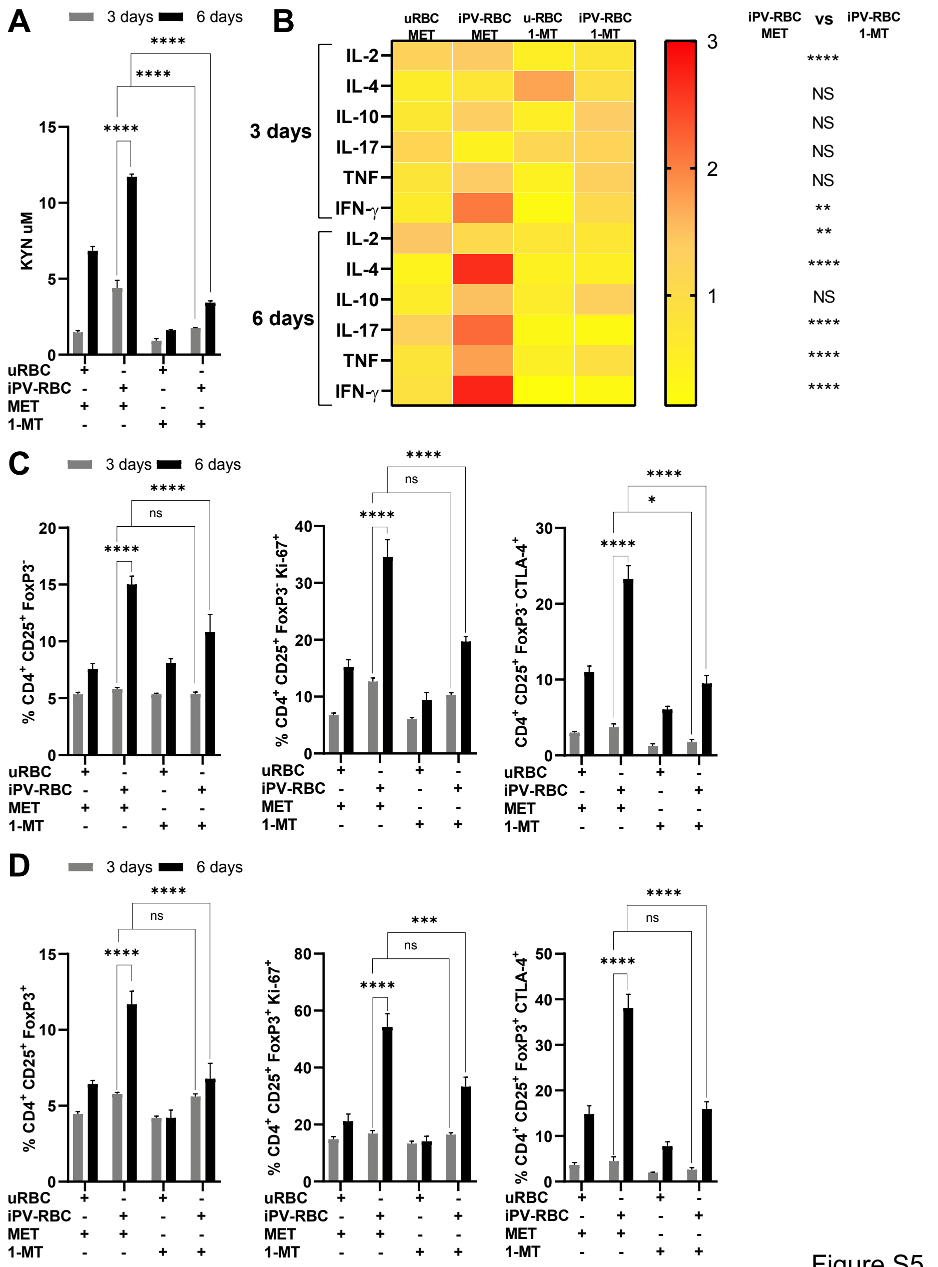
A
3 days
6 days
KYN uM
uRBC
iPV-RBC
MET
1-MT
B
uRBC MET
iPV-RBC MET
u-RBC 1-MT
iPV-RBC 1-MT
3 days
6 days
IL-2
IL-4
IL-10
IL-17
TNF
IFN-γ
iPV-RBC MET vs iPV-RBC 1-MT
****
NS
NS
NS
NS
**
**
****
NS
****
****
****
C
% CD4+ CD25+ FoxP3-
% CD4+ CD25+ FoxP3- Ki-67+
CD4+ CD25+ FoxP3- CTLA-4+
ns
*
D
% CD4+ CD25+ FoxP3+
% CD4+ CD25+ FoxP3+ Ki-67+
% CD4+ CD25+ FoxP3+ CTLA-4+
***

Figure S5

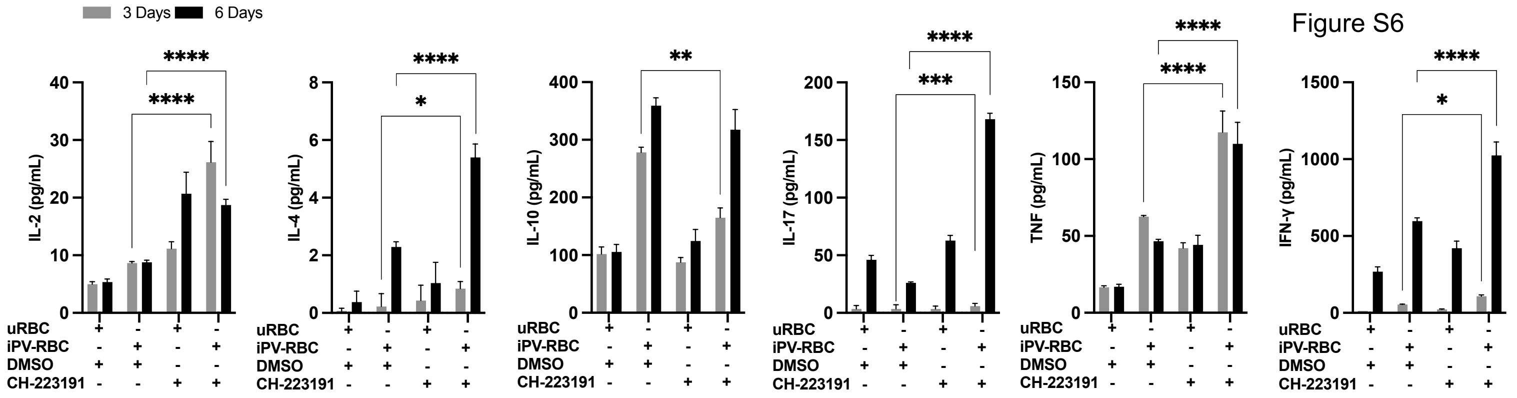

3 Days
6 Days
IL-2 (pg/mL)
IL-4 (pg/mL)
IL-10 (pg/mL)
IL-17 (pg/mL)
TNF (pg/mL)
IFN-γ (pg/mL)
uRBC
iPV-RBC
DMSO
CH-223191

Figure S6

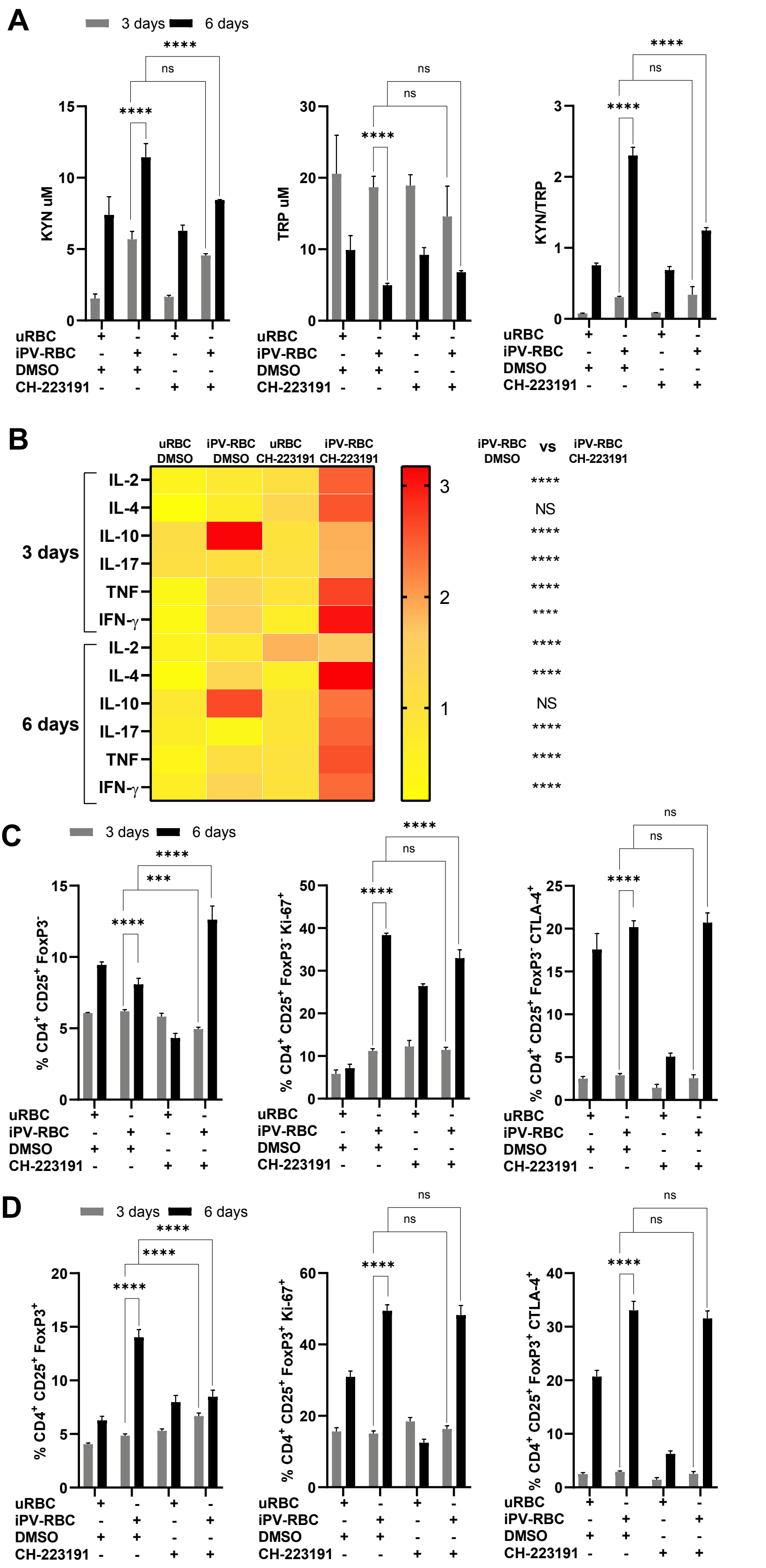
A
3 days
6 days
KYN uM
TRP uM
KYN/TRP
uRBC
iPV-RBC
DMSO
CH-223191
ns
****
B
uRBC DMSO
iPV-RBC DMSO
uRBC CH-223191
iPV-RBC CH-223191
iPV-RBC DMSO vs iPV-RBC CH-223191
3 days
6 days
IL-2
IL-4
IL-10
IL-17
TNF
IFN-γ
NS
C
% CD4+ CD25+ FoxP3-
% CD4+ CD25+ FoxP3- Ki-67+
% CD4+ CD25+ FoxP3- CTLA-4+
***
D
% CD4+ CD25+ FoxP3+
% CD4+ CD25+ FoxP3+ Ki-67+
% CD4+ CD25+ FoxP3+ CTLA-4+

Figure S7

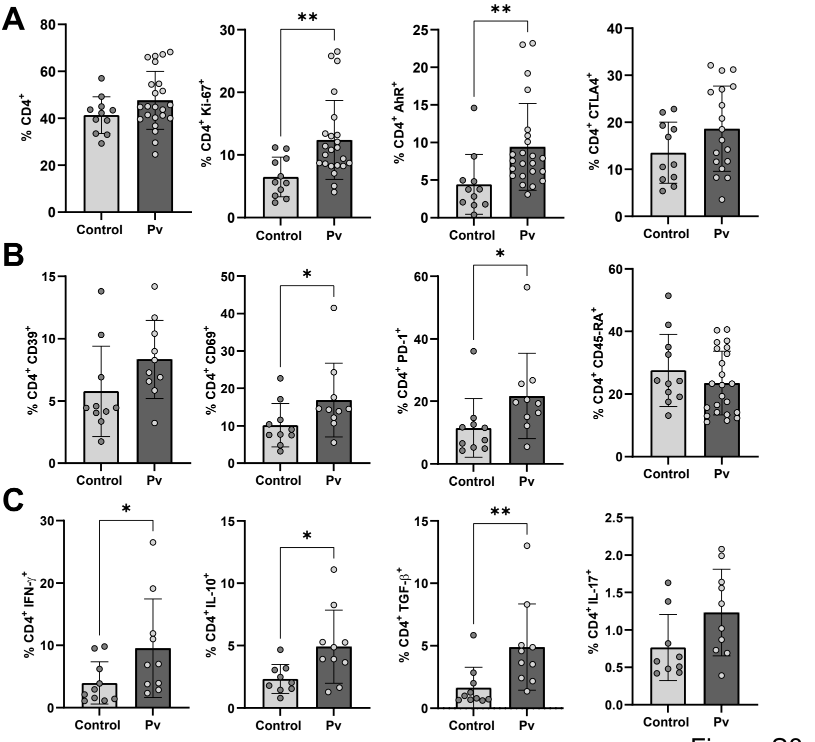
A
B
C
% CD4+
% CD4+ Ki-67+
% CD4+ AhR+
% CD4+ CTLA4+
% CD4+ CD39+
% CD4+ CD69+
% CD4+ PD-1+
% CD4+ CD45-RA+
% CD4+ IFN-γ+
% CD4+ IL-10+
% CD4+ TGF-β+
% CD4+ IL-17+
Control
Pv
**
*

Figure S8

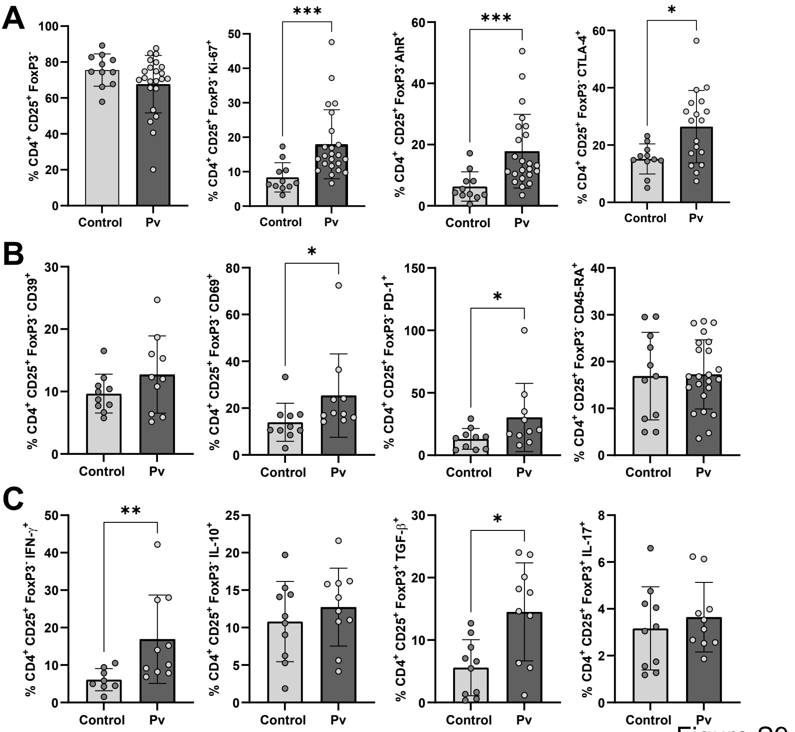

A
B
C
% CD4+ CD25+ FoxP3-
% CD4+ CD25+ FoxP3- Ki-67+
% CD4+ CD25+ FoxP3- AhR+
% CD4+ CD25+ FoxP3- CTLA-4+
% CD4+ CD25+ FoxP3- CD39+
% CD4+ CD25+ FoxP3- CD69+
% CD4+ CD25+ FoxP3- PD-1+
% CD4+ CD25+ FoxP3- CD45-RA+
% CD4+ CD25+ FoxP3- IFN-γ+
% CD4+ CD25+ FoxP3- IL-10+
% CD4+ CD25+ FoxP3+ TGF-β+
% CD4+ CD25+ FoxP3+ IL-17+
Control
Pv
***
*
**

Figure S9

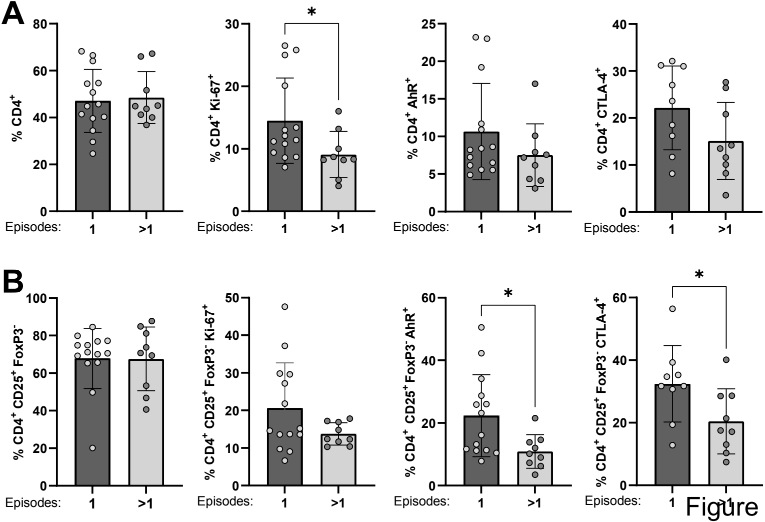
A
B
% CD4+
% CD4+ Ki-67+
% CD4+ AhR+
% CD4+ CTLA-4+
% CD4+ CD25+ FoxP3-
% CD4+ CD25+ FoxP3- Ki-67+
% CD4+ CD25+ FoxP3- AhR+
% CD4+ CD25+ FoxP3- CTLA-4+
Episodes:
1
>1
*

Figure S10

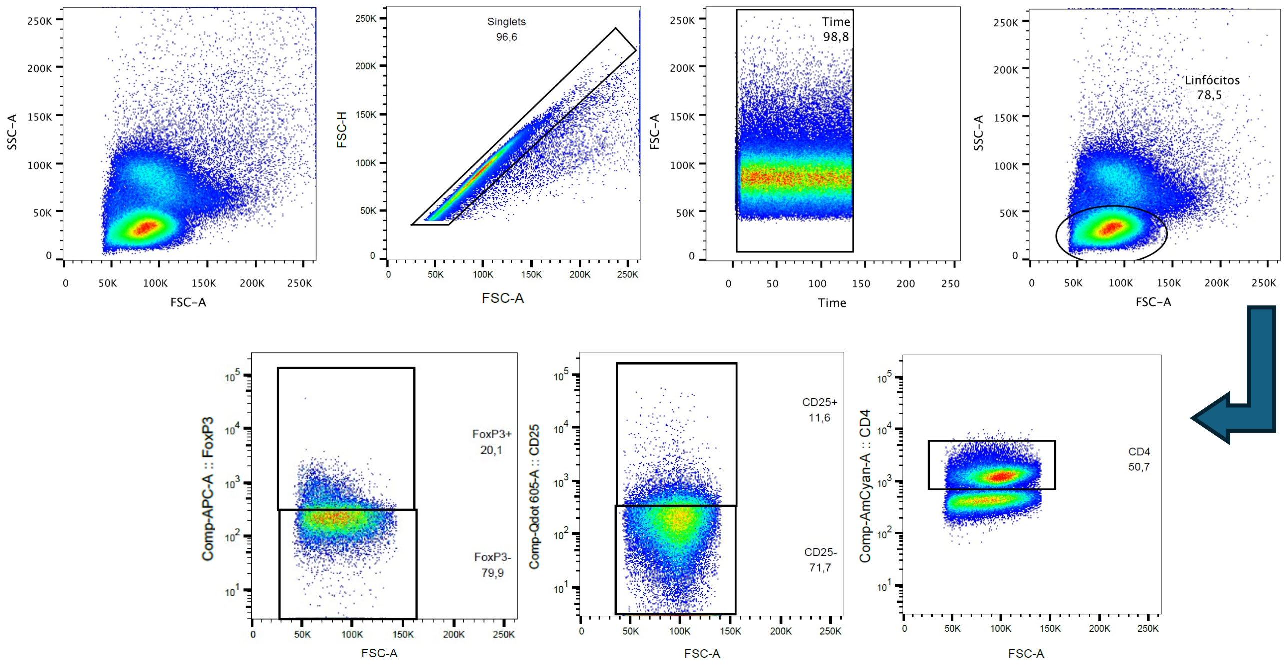
SSC-A
FSC-A
Singlets
96,6
FSC-H
Time
98,8
Linfócitos
78,5
Comp-APC-A :: FoxP3
FoxP3+
20,1
FoxP3-
79,9
Comp-Qdot 605-A :: CD25
CD25+
11,6
CD25-
71,7
Comp-AmCyan-A :: CD4
CD4
50,7

Figure S11

Analysis of FMO Data Sets Controls

CD4+ T cells

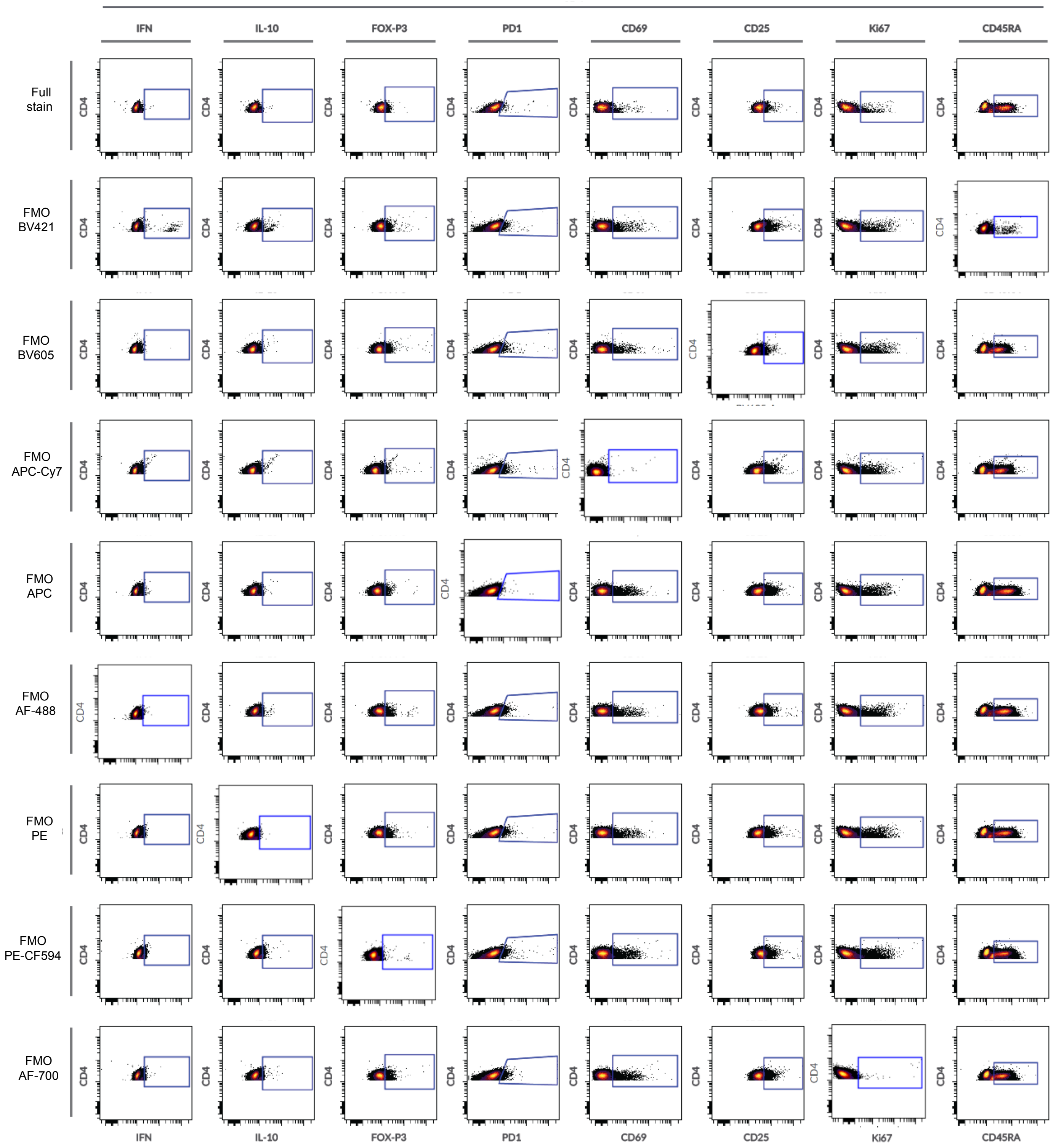

Figure S12

Supplementary Table 1: Flow cytometry markers

Panel	Antibody	Fluorochrome	Catalog	Clone	Mark
**Treg (Patients)**	CD4	BV 510	317444	OKT4	BioLegend
	CD25	BV 605	302632	BC96	BioLegend
	FoxP3	AF647	560045	259D/C7	BD
	CD45RA	PerCP-Cy5.5	563429	HI100	BD
	CTLA4	PE-CF594	562742	BNI3	BD
	CD69	BV421	562884	FN50	BD
	PD1	BV650	564324	MIH4	BD
	CD39	PE	555464	TU66	BD
	Ki-67	Pe-Cy7	12-5699-42	B56	BD
	IFN-γ	BC421	562988	B27	BD
	IL-10	BV650	564051	JES3-9D7	BD
	TGF-β	PE-CF594	562422	TW4-9E7	BD
	IL-17	Alexa700	560613	N49-653	BD
	AhR	Primary	14-9854-82	FF3399	eBioscience
	Secondary	FITC	-	-	BD
**Treg (*In Vitro*)**	CD4	APC-H7	560158	RPA-T4	BD
	CD25	BB515	564467	2A3	BD
	FoxP3	PE-Cy7	25-4777-42	236A/E7	eBioscience
	Ki-67	PE	350504	Ki-67	BioLegend
	CTLA-4	APC	555855	BNI3	BD
	CD45-RA	PerCP-Cy5.5	563429	HI100	BD
**IDO1 (*In vitro*)**	CD14	PE	555398	M5E2	BD
	HLA-DR	APC	557344	G46-6	BD
	IDO1	PerCP-eFluor 710	46-9477-42	eyedio	eBioscience

Supplementary Table 2: Primers used in qPCR

Target	Forward	Tm (ºC)	Reverse	Tm (ºC)
IDO1	GCCTGATCTCATAGAGTCTGGC	56.7	TGCATCCCAGAACTAGACGTGC	56.7
IDO2	GTTATGTCTGGCAGGAAGGAGAG	57.1	GTCCAGTTCGTCAGCACCAAGT	56.7
GAPDH	GTCTCCTCTGACTTCAACAGCG	56.7	ACCACCCTGTTGCTGTAGCCAA	56.7
ACTB	CACCATTGGCAATGAGCGGTTC	56.7	AGGTCTTTGCGGATGTCCACGT	56.7
B2M	CCACTGAAAAAGATGAGTATGCCT	54	CCAATCCAAATGCGGCATCTTCA	55.3

Supplementary Table 3: Analysis of FMO Data Sets Controls on CD4$^+$ T cells

Staining	AF-488	PE	PE-CF594	PerCP	APC	AF-700	APC-CY7	BV421	BV605
Full	IFN-γ	IL-10	FOXP3	CD4	PD-1	Ki67	CD69	CD45RA	CD25
FMO BV421	IFN-γ	IL-10	FOXP3	CD4	PD-1	Ki67	CD69		CD25
FMO BV605	IFN-γ	IL-10	FOXP3	CD4	PD-1	Ki67	CD69	CD45RA	
FMO APC-Cy7	IFN-γ	IL-10	FOXP3	CD4	PD-1	Ki67		CD45RA	CD25
FMO APC	IFN-γ	IL-10	FOXP3	CD4		Ki67	CD69	CD45RA	CD25
FMO AF-488		IL-10	FOXP3	CD4	PD-1	Ki67	CD69	CD45RA	CD25
FMO PE	IFN-γ		FOXP3	CD4	PD-1	Ki67	CD69	CD45RA	CD25
FMO PE-CF594	IFN-γ	IL-10		CD4	PD-1	Ki67	CD69	CD45RA	CD25
FMO AF-700	IFN-γ	IL-10	FOXP3	CD4	PD-1		CD69	CD45RA	CD25
